# CUL4B-DDB1-COP1-mediated UTX downregulation promotes colorectal cancer progression

**DOI:** 10.1186/s40164-023-00440-z

**Published:** 2023-09-07

**Authors:** Dakui Luo, Min Chen, Qingguo Li, Kangjunjie Wang, Kaihua Wang, Junqiang Li, Guoxiang Fu, Zezhi Shan, Qi Liu, Yufei Yang, Lei Liang, Yanlei Ma, Yi Qin, Jun Qin, Daming Gao, Xinxiang Li

**Affiliations:** 1 Department of Colorectal Surgery, Fudan University Shanghai Cancer Center, Shanghai, 200032 China; 2 Department of Oncology, Shanghai Medical College, Fudan University, Shanghai, 200032 China; 3 State Key Laboratory of Cell Biology, Shanghai Institute of Biochemistry and Cell Biology, Center for Excellence in Molecular Cell Science, Chinese Academy of Sciences, Shanghai, 200031 China; 4 University of Chinese Academy of Sciences, Beijing, 100049 China; 5 D1 Medical Technology (Shanghai) Co., Ltd, Shanghai, 201802 China; 6 Department of Pancreatic Surgery, Fudan University Shanghai Cancer Center, Shanghai, 200032 China; 7 CAS Key Laboratory of Tissue Microenvironment and Tumor, Shanghai Institute of Nutrition and Health, Chinese Academy of Sciences, 320 Yueyang Road, Shanghai, 200031 China; 8 School of Life Science, Hangzhou Institute for Advanced Study, University of Chinese Academy of Sciences, Hangzhou, 310024 China

**Keywords:** Colorectal cancer, COP1, Ubiquitylation, UTX

## Abstract

**Background:**

UTX (encoded by *KDM6A*), a histone demethylase for H3K27me2/3, is frequently mutated in human cancers. However, its functional and regulatory mechanisms in colorectal cancer (CRC) remain unclear.

**Methods:**

Immunohistochemistry staining was used to investigate the clinical relevance of UTX in CRC. Additionally, we generated a spontaneous mouse CRC model with conditional *Utx* knockout to explore the role of UTX in the colorectal tumorigenesis. Post-translational regulation of UTX was determined by co-immunoprecipitation and immunoblot analyses.

**Results:**

Herein, we identify that downregulation of UTX, mediated by the Cullin 4B-DNA Damage Binding Protein-1-Constitutive Photomorphogenesis Protein 1 (CUL4B-DDB1-COP1) complex, promotes CRC progression. *Utx* deletion in intestinal epithelial cells enhanced the susceptibility to tumorigenesis in AOM/DSS-induced spontaneous mouse CRC model. However, this effect is primarily alleviated by GSK126, an inhibitor of histone methyltransferase EZH2. Mechanistically, *EMP1* and *AUTS2* are identified as putative UTX target genes mediating UTX functions in limiting intestinal tumorigenesis. Notably, the CUL4B-DDB1-COP1 complex is identified as the functional E3 ligase responsible for targeting UTX for degradation in CRC cells. Thus, *Cop1* deficiency in mouse intestinal tissue results in UTX accumulation and restricts tumorigenesis. Furthermore, patient cohort analysis reveals that UTX expression is negatively correlated with clinical stage, favorable disease outcomes, and COP1 expression.

**Conclusions:**

In the current study, the tumor suppressor function and regulation of UTX in CRC provide a molecular basis and the rationale to target EZH2 in UTX-deficient CRC.

**Supplementary Information:**

The online version contains supplementary material available at 10.1186/s40164-023-00440-z.

## Background

Colorectal cancer (CRC) is the third most commonly diagnosed cancer and the second cause of cancer-related deaths among 36 cancer types globally in 2020 [1]. The initiation and progression of CRC are commonly regarded as a complicated and heterogeneous process involving genetic mutations [2] and dysregulated epigenetic modifications [3, 4]. Histone methylation, a reversible modification associated with gene activation or silencing, is regulated by specific methyltransferases and demethylases. Global changes in histone methylation have been well-established in carcinogenesis and identified as prognostic biomarkers in a spectrum of human tumors, including CRC [5–8].

The ubiquitously transcribed tetratricopeptide repeat on the X chromosome (UTX, encoded by *KDM6A* located on the X chromosome) is a histone demethylase that catalyzes the removal of H3K27me2/3, promoting the transcription of target genes. UTX is involved in a variety of biological processes, comprising homeotic gene expression, embryonic development, and cellular reprogramming [9]. UTY, a paralog sharing 83% sequence similarity with UTX, lacks detectable histone demethylase activity [10]. Interestingly, the loss of UTX during the development could be compensated by UTY, suggesting the function of UTX during embryonic development might be independent of its demethylase activity between E9.5 and E13.5 [11]. Recent studies have identified UTX as a highly mutated tumor suppressor, with reduced expression observed in several human malignancies [12–17]. It has also been recognized as an “escape from X-inactivation tumor suppressor”, with a distinct dosage effect of *UTX* copy number observed during tumorigenesis [17]. The mutation or deletion of both copies of *UTX* is required for the dysfunction of this tumor suppressor in females, while loss of one copy of *UTX* achieves the same effect in males. UTY provides limited compensation for UTX loss during tumorigenesis, suggesting that the tumor suppressor function of UTX is demethylase activity-dependent. Accumulating evidence indicated that the oncogenic effect of UTX loss is mediated mainly through an increase in the EZH2 level in lung cancer [16], and UTX loss sensitizes cells to EZH2 inhibition in multiple myeloma [18]. Although a previous report suggested that UTX promotes CRC progression [19], the functional and regulatory mechanisms of UTX in CRC is largely unclear.

The ubiquitin–proteasome system is a significant pathway that precisely governs protein stability [20]. Among the Cullin-RING Ligase (CRL) family [21], a typical CRL4 ligase is composed of a scaffold Cullin (CUL4A or CUL4B) protein, a linker protein-DNA Damage Binding Protein-1 (DDB1), a substrate recognition adaptor (DCAF), and a ring protein with intrinsic E3 activity (RBX1 or RBX2) [22]. The substrate specificity of CRL4 is mainly dependent on the DCAF protein loaded on the specific CRL4 complex. Constitutive photomorphogenic 1 (COP1, also known as RFWD2) was initially identified as a negative regulator of photomorphogenesis in *Arabidopsis* [23], and its functions have been extensively studied in the context of light signaling in plants [24]. Notably, mammalian COP1 possesses intrinsic E3 ubiquitin ligase activity, enabling it to either promote protein ubiquitination or serve as a DCAF protein that forms a CRL4 ligase complex to ubiquitinate substrates [25]. Intriguingly, both classic oncoprotein and tumor suppressor proteins, such as c-Jun, ETS transcription factors, and P53, have been identified as ubiquitination-degradation substrates of COP1, indicating a complex and context-dependent role of COP1 in cancer [26–28]. However, the role of COP1 in CRC has not yet been reported.

In the current study, we found that UTX is negatively correlated with clinical stage, and low UTX expression is associated with poor patient survival. Subsequently, we validated the tumor suppressor role of UTX in CRC by specifically depleting the *Utx* gene in intestinal epithelial cells and inducing de novo colorectal tumorigenesis. Our mechanistic study showed that UTX loss promoted colorectal oncogenesis partially via transcriptionally repression of *EMP1* and *AUTS2*. Experiments performed with mouse CRC model and human CRC organoids further suggested that UTX deficiency confers enhanced sensitivity to EZH2 inhibitor GSK126 in CRC. Next, we certified that UTX is subject to CRL4-COP1 complex-mediated degradation, partially explaining the oncogenic role of COP1 in CRC.

## Methods

### Human CRC tissue microarray (TMA) construction

A human TMA was obtained from CRC patients who underwent radical surgery between January 2007 and November 2009 at Fudan University Shanghai Cancer Center (FUSCC), Shanghai, China. Human CRC TMA was carried out as described previously [29]. The clinicopathological characteristics of patients, such as age, gender, tumor location, histological type, tumor-node-metastasis (TNM) stage, grade, venous and nervous invasion, pretreatment serum CEA level, and survival data, were collected from the FUSCC database.

### Generation of genetically-modified mice

All mice were raised in a specific pathogen-free environment at the Shanghai Institute of Biochemistry and Cell Biology. *Utx*^f/f^ and *Villin*-Cre mice were provided by Dr. Charlie Degui Chen and Dr. Anning Lin (Shanghai Institute of Biochemistry and Cell Biology, CAS), respectively. As described previously [15], the exons 11 to 14 of *Utx* were deleted using a targeting vector, which disrupts the *Utx* H3K27 demethylase domain by triggering a frameshift mutation. *Cop1*^f/f^ mice were purchased from Laboratory Animal Center, East China Normal University. *Utx*^−/y^ or *Utx*^−/+^ mice were generated by crossing *Utx*^f/f^ mice with *Villin*-Cre mice, while *Utx*^−/−^ mice were embryonic lethal. *Cop1*^−/+^ mice were generated by crossing *Cop1*^f/f^ mice with *Villin*-Cre mice, while *Cop1*^−/−^ mice were embryonic lethal. All mice were maintained in a C57BL/6 background. The primers for genotyping are listed in Additional file 1: Table S1.

### Induction of colitis, CRC, and treatments

We combined the carcinogen azoxymethane (AOM) treatment with repeated administration of dextran sodium sulfate (DSS) in drinking water to induce colorectal tumors. wild-type (WT), *Utx*^−/y^, and *Utx*^−/+^ mice were used for AOM/DSS treatment. 8–10-week-old mice were injected intraperitoneally with AOM (10 mg/kg, Sigma, A5486). After one week, DSS was solubilized into the drinking water to feed mice for 5 days (2%, MP Biomedicals, 160,110). Subsequently, regular drinking water was given for 14 days. The DSS treatment was repeated for another two cycles. Body weights were recorded during the DSS treatment. The mice were sacrificed 90 days after the AOM injection, and colorectums were obtained for further analysis. Gross inspection, total tumor number, tumor burden, and tumor size were recorded. The samples were fixed as ‘‘swiss-rolls,’’ and a histopathological examination was conducted.

For drug treatment, 2 weeks after AOM/DSS treatment, WT and *Utx*^*−/y*^ mice were randomized into two groups. Then, the mice were treated with GSK126 (50 mg/kg, dissolved in SBE-β-CD) or vehicle control daily for additional 2 weeks before being sacrificed for analysis.

### Immunoblotting (IB) and immunoprecipitation (IP)

Cells were lysed using EBC lysis buffer (50 mM Tris–HCl, pH 8.0, 120 mM NaCl, 0.5% Nonidet P-40) with protease inhibitors (1:100, Selleck) and phosphatase inhibitors (1:100, Selleck). Intestinal/tumor tissues from human or mice were lysed using Minute™ Total Protein Extraction Kit (Invent Biotechnologies, Inc.) with protease inhibitors (1:100, Selleck) and phosphatase inhibitors (1:100, Selleck). The protein concentrations of lysates were determined using the Bio-Rad protein assay kit on a spectrophotometer (Thermo Scientific). An equivalent of 30 μg total lysate from each sample was used for IB. The cell lysates were incubated with anti-FLAG M2 agarose beads or anti-HA agarose beads for 2–4 h for IP. The pellets were washed with NETN buffer (20 mM Tris–HCl, pH 8.0, 100 mM NaCl, 0.5% Nonidet P-40, 1 mM EDTA) before analysis by SDS-PAGE and IB with specific antibodies.

### Public database analysis

Datasets from The Cancer Genome Atlas (TCGA) (https://portal.gdc.cancer.gov), Genotype-tissue expression (GTEx) (https://www.gtexportal.org/home/index.html) and the Gene Expression Omnibus (GEO, https://www.ncbi.nlm.nih.gov/sites/GDSbrowser?acc=GDS2947) were utilized to determine *COP1* mRNA expression in colon cancer, colorectal adenomas and normal colon tissues.

### Immunohistochemistry (IHC)

IHC staining was performed with indicated antibodies using the standard protocol. The TMA was incubated with an anti-UTX antibody (1:400 dilution, 33510S, Cell Signaling Technology) and an anti-COP1 antibody (1:100 dilution, A300-894A, Bethyl). Mouse tissues were incubated with anti-UTX antibody (1:100 dilution, GTX121246, GeneTex), anti-COP1 antibody (1:100 dilution, A300-894A, Bethyl), Ki67 (1:100 dilution, ab15580, Abcam), anti-EZH2 antibody (1:200 dilution, ab191080, Abcam), and anti-H3K27me3 antibody (1:100 dilution, ab192985, Abcam; 1:100 dilution, A2363, Abclonal), respectively, as indicated. The IHC stained tissue sections were scored by two independent pathologists blinded to the clinicopathological features. The proportion of staining was graded as 0 (< 5%), 1 (5–25%), 2 (26–50%), 3 (51–75%), and 4 (> 75%) based on the percentages of the positive staining areas *vs*. the whole area. The staining intensity was graded as 0 (negative), 1 (weak), 2 (medium,) or 3 (strong). The immunoreactivity score (IRS) was calculated by multiplying the score of staining proportion with staining intensity. IRS ≤ 4 was considered as low, and > 4 was considered as high.

### Cell proliferation, colony formation, and soft agar colony formation assays

HCT116 cells were seeded into 96-well plates at a density of 3000 cells/well. Then, the cells were incubated with CCK-8 reagent (C0039, Beyotime Biotechnology, Shanghai, China) for 2 h, and absorbance was measured at 450 nm. For colony formation assay, HCT116 cells were seeded in the 96-well plates with 500 cells/well and incubated for 10 days. For the soft agar colony formation assay, 6-well plates were covered with lower agar (1.5 mL, 0.8%), and 500 cells were seeded in the upper agar (1.5 mL, 0.4%). After 2 weeks, the colonies were stained with 1% crystal violet (Beyotime).

### ChIP-seq assay and data analysis

The distal colorectum samples of 2-month-old WT and *Utx*^−/y^ mice (each sample mixed with three animals) were collected for ChIP-seq analysis service by Active Motif Inc. using the antibody against H3K27me3 (Active Motif). The data were deposited in the NCBI’s SRA (Accession: PRJNA752794).

### RNA-seq analysis

The distal colorectum mRNAs from 2-month-old WT mice and *Utx*^−/y^ mice (each RNA sample mixed with three animals) were obtained for transcriptome analysis service by Sangon Biotech. The data were deposited in the NCBI’s SRA (Accession: PRJNA734666).

### siRNA screening for putative target genes

siRNA-based screening was performed for the selected 55 genes in HCT116 cells. The siRNAs were selected from Dharmacon Human ON-TARGET plus siRNA Library. To eliminate the off-target effects, four independent siRNAs (SMARTpools) were used for each gene. siNC and si*TOX* were selected as the negative and positive controls in the screening assay. 0.15 μL lipofectamine RNAiMAX reagent (Thermo) was added to the siRNA-loaded 96-well plates using Multidrop Combi (Thermo). Then, HCT116 cells were seeded in the 96-well plates at a density of 1500 cells/well. The number of cells was counted in the following 4 days using Ensight (Perkin Elmer). Subsequently, CTG (G756A, Promega) was added and measured on an Envision plate reader (Perkin Elmer).

### Total RNA extraction and quantitative PCR

Total RNA was extracted from cells or tissues using TRIzol (Invitrogen) according to the manufacturer’s instructions. The cDNA was synthesized using ABScript II RT Master Mix (ABclonal Technology). Quantitative PCR was performed using a SYBR.

Kit (Takara) according to the manufacturer’s protocol. Human *GAPDH* or mouse *β*-*Actin* was used as an internal control. The primer sequences are listed in Additional file 1: Table S1.

### ChIP-qPCR assays

The ChIP assay was performed using truChIP Chromatin Shearing Kit (Covaris) and EZ-ChIP kit (Millipore). The procedure was according to the kit instruction manual provided by the manufacturer. Primer sequences can be found in Additional file 1: Table S1.

### Plasmid construction

HA-UTX and different mutants were cloned into pcDNA3.1 vector. FLAG-UTX was generated by inserting *UTX* cDNA into pCMV-FLAG and pRLenti-CMV-MCS-3FLAG-PGK-Puro vectors. The Flag-tagged of DET1, DDB1, RBX1, Cullin1, Cullin2, Cullin3, Cullin4A, Cullin4B, and Cullin5 were generated by inserting cDNAs into pCMV-FLAG vector. The Flag-tagged and HA-tagged coding sequence of human *COP1* WT or the relevant isoforms and the mutants were inserted into the lentiviral vector pLex-MCS-CMV-puro (Addgene, USA) to generate COP1 expression plasmids. Myc-Ub was provided by Dr. Cory Ronggui Hu (Shanghai Institute of Biochemistry and Cell Biology, CAS). pLKO.1-TRC cloning vector (Addgene: 10,878) was used to express shRNAs against *COP1* or *UTX*. Briefly, the oligos targeting *COP1*, *UTX, CUL1* and *CUL4B* were cloned into the pLKO.1-TRC vector to generate shRNA constructs.

### Cell culture and transfection

HCT116, HT-29, SW620, SW480, LoVo, DLD1, and HEK293T cells were kindly provided by Cell Bank, Chinese Academy of Science and were cultured in DMEM containing 10% FBS (Invitrogen) at 37 °C in 5% CO_2_. Plasmids were transiently transfected with polyethylenimine (Sigma) or Lipofectamine 3000 (Invitrogen). Cells were transfected with siRNA using Lipofectamine 3000 (Invitrogen) according to the manufacturer’s protocol.

The siRNAs targeting indicated genes were synthesized by Biotend (Shanghai, China), and the sequences are listed in Additional file 1: Table S2.

### Human primary CRC organoid culture

Organoids were cultured in Advanced DMEM/F12 medium (GIBCO, 12,634–010), containing 500 ng/mL R-spondin 1 (Sino Biological, 11,083-HNAS), 100 ng/mL Noggin (Sino Biological, 50,688-M02H), 50 ng/mL EGF (Sino Biological, 50,482-MNCH), 100 ng/mL FGF-10 (Sino Biological, 10,573-HNAE), 1X HEPES (GIBCO, 15,630,080), 1X Glutamax (GIBCO, 35,050,061), 100 μg/mL Normocin (InvivoGen, ant-nr-1), 1X Gentamicin/amphotericin B (GIBCO, R01510), 1X B27 (Invitrogen, 17,504–044), 1X N2 (Invitrogen, 17,504–048), 1 mM n-Acetylcysteine (Sigma-Aldrich, A9165), 10 mM nicotinamide (Sigma-Aldrich, N0636), 0.5 μM A-83–01 (Tocris, 2939), 10 μM Y-27632 (Sigma-Aldrich, Y0503), 10 nM Gastrin, 1 μM prostaglandin E2 (Sigma-Aldrich, P6532), and 3 μM SB202190 (Sigma-Aldrich, S7067). The culture medium was refreshed every 3 days.

### Organoid cytotoxicity assay

Organoids were harvested, passaged, and seeded in a 48-well cell culture plate. Each well contained about 100 ± 50 organoids in 20 μL Matrigel with 300 μL culture medium. Once the organoids grew to 100 μm in diameter, the organoids culture medium was replaced with 300 μL drug-containing culture medium with GSK126. The drug-containing culture medium was refreshed every 3 days. Organoids were photographed every 3 days during the 9-day drug treatment period. The size of live organoids was measured using ImageJ 1.53a (National Institutes of Health, USA).

### TdT-UTP nick end labeling (TUNEL) assay

TUNEL assay was performed with a colorimetric TUNEL apoptosis assay kit according to the manufacturer’s instructions (C1091, Beyotime).

### 5-ethynyl-2’-deoxyuridine (EdU) assay

EdU assay was conducted using BeyoClick™ EdU Cell Proliferation Kit with DAB (C0085S, Beyotime).

### Xenograft tumorigenesis assay

HCT116 cells expressing ectopic COP1, ectopic COP1 plus UTX mutant (V607A/P608A/V1205A/P1206A), and empty vector were injected into the flank of each nude mouse (1 × 10^7^ cells each mouse) to evaluate the tumorigenesis. Tumor volumes were measured and recorded every 3 days.

### Antibodies and chemical inhibitors

Primary antibodies used in this study are as follows: UTX (33510S, Cell Signaling Technology; GTX121246, GeneTex), COP1 (A300-894A, Bethyl), CUL1 (SC-17775, Santa Cruz Biotechnology), CUL2 (A5308, Abclonal), CUL3 (2759, Cell Signaling Technology), CUL4A (AB60215a, Abgent), CUL4B (A6198, Abclonal), CUL5 (SC-373822, Santa Cruz Biotechnology), EMP1 (ab230445 and ab202975, Abcam), AUTS2 (25,001–1-AP, Proteintech), Ki67 (ab15580, Abcam), EZH2 (ab191080, Abcam), VEGFC (A2556, Abclonal), Caspase-9 (A0281, Abclonal), Bcl2 (15,071, Cell Signaling Technology), DDB2 (ab181136, Abcam), DCAF7 (ab138490, Abcam), DCAF13 (ab195121, Abcam), H3K27me3 (ab192985, Abcam; A2363, Abclonal), H3 (ab1791, Abcam), P27 (610,241, BD), HA-Tag (51,064–2-AP, Proteintech; SC-7392, Santa Cruz Biotechnology), Flag-Tag (F7425-0.2MG, Sigma; F3165-1MG, Sigma), GFP (66,002–1-Ig, Proteintech), Myc-Tag (16,286–1-AP, Proteintech), α-tubulin (SC-23948, Santa Cruz Biotechnology), and Vinculin (V4505, Sigma). The chemical inhibitors MG132 (S2619), MG101 (S7386), MLN4924 (S7109), Baf-A1 (S1413), and GSK126 (S7061) were purchased from Selleck.

### Statistical analysis

Statistical evaluation was conducted using SPSS 25.0 (SPSS Inc., Chicago, IL, USA) and GraphPad Prism v.8 (La Jolla, CA, USA). The experimental data were analyzed using a two-tailed Student’s t-test, one-way analysis of variance (ANOVA) with Dunnett’s multiple comparisons test, Pearson’s correlation coefficients test, and log-rank test. P < 0.05 was considered significant.

## Results

### Low UTX expression predicts poor survival in human CRC

**Fig. 1:**
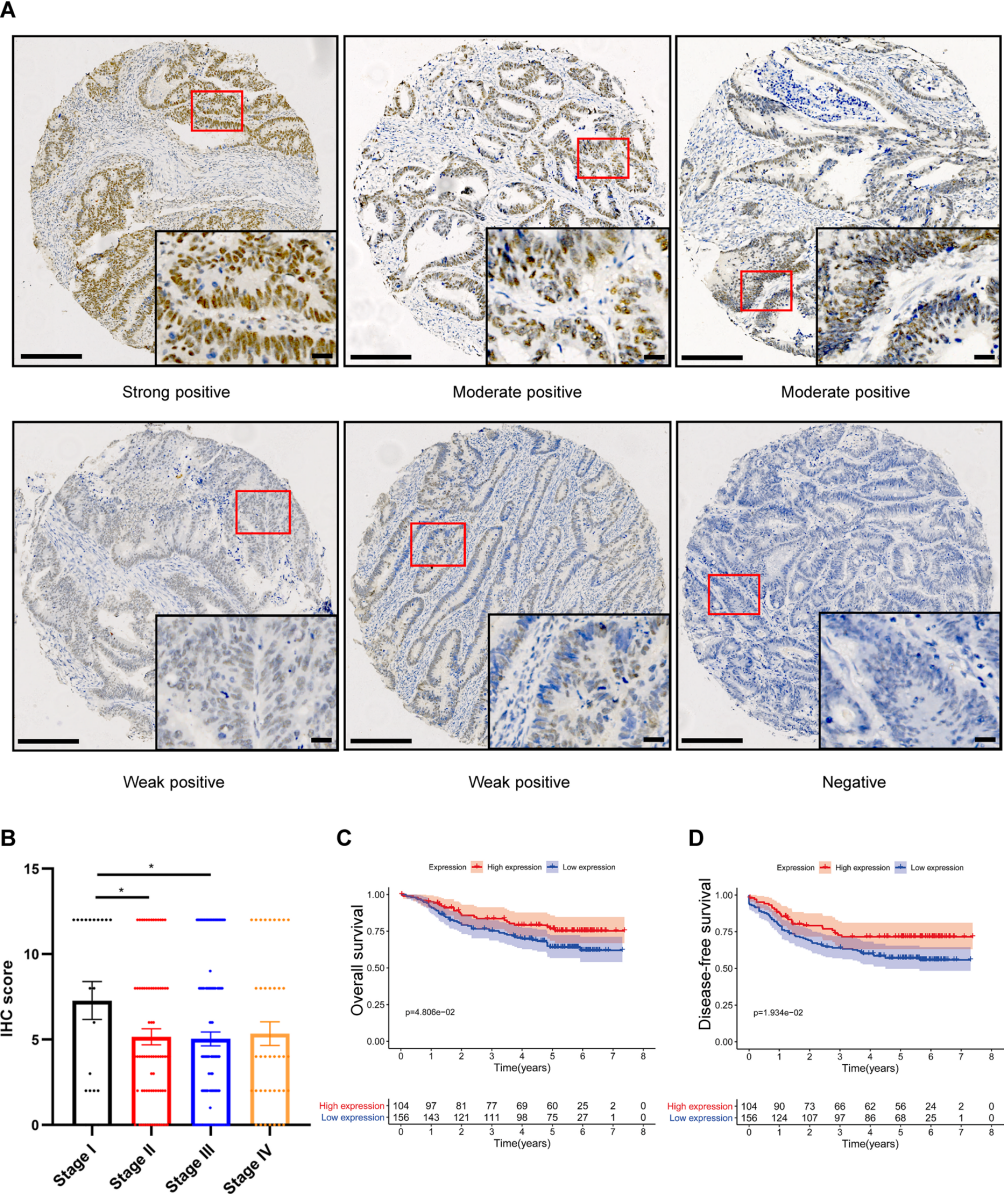
UTX expression is associated with CRC progression. **A** Representative IHC images of UTX staining in CRC patients. Scale bar, 200 μm (left) and 20 μm (right). The proportion of staining was graded as 0 (< 5%), 1 (5–25%), 2 (26–50%), 3 (51–75%), and 4 (> 75%) based on the percentages of the positive staining areas *vs*. the whole area. The staining intensity was graded as 0 (negative), 1 (weak), 2 (medium), or 3 (strong). The IRS was calculated by multiplying the score of staining proportion and intensity. IRS ≤ 4 was considered low, and > 4 was considered high. IRS of representative images (upper-left, upper-middle, upper-right, bottom-left, bottom-middle, bottom-right): 12, 8, 6, 4, 2, and 0. The proportion of staining: 90%, 90%, 70%, 90%, 50%, and 0%. Staining intensity: 3, 2, 2, 1, 1, and 0. **B** UTX protein expression assessed by IHC scores of TMA at different pathological stages (stage I: n = 21, stage II: n = 76, stage III: n = 123, stage IV: n = 40). **C** Kaplan–Meier plot of OS of CRC patients based on UTX expression. **D** Kaplan–Meier plot of DFS of CRC patients based on UTX expression. Data information: In (B), data are presented as mean ± SEM, ^*^P < 0.05 (two-tailed Student’s t-test). In (C and D), log-rank test

To investigate the putative role of UTX in CRC, a TMA containing 260 CRC samples was generated to examine UTX expression. Immunochemistry (IHC) staining results indicated significantly lower UTX expression in stages II and III CRC patients than in stage I (Fig. 1A and B). Specifically, low UTX expression was commonly observed in male patients (P = 0.003) and patients with advanced T stage disease (P = 0.045) (Table 1). Importantly, low UTX expression was significantly associated with poor overall survival (OS) (P = 0.048, Fig. 1C) and disease-free survival (DFS) (P = 0.019, Fig. 1D) in the current CRC cohort. Multivariable Cox regression analysis further revealed that UTX expression is an independent predictor for risk stratification of DFS in CRC patients (P = 0.037, Tables 2 and 3). Therefore, low UTX expression is associated with advanced CRC stages and unfavorable clinical outcomes, which is consistent with the tumor suppressor role of UTX reported in other cancer types [16, 17].

**Table 1:** Clinicopathological characteristics of CRC patients according to UTX expression

	UTX expression		
	High group (n = 104), %	Low group (n = 156), %	P valuve
Characteristics			
Age			0.325
≤ 60	67 (42.4)	91 (57.6)	
> 60	37 (36.3)	65 (63.7)	
Gender			**0.003**
Male	52 (32.7)	107 (67.3)	
Female	52 (51.5)	49 (48.5)	
Tumor Location			0.085
Colon	42 (34.4)	80 (65.6)	
Rectum	62 (44.9)	76 (55.1)	
Preoperative Serum CEA			0.916
Elevated	36 (40.0)	54 (60.0)	
Normal	65 (40.4)	96 (59.6)	
Unknown	3 (33.3)	6 (66.7)	
pT stage			**0.045**
2	23 (56.1)	18 (43.9)	
3	23 (42.6)	31 (57.4)	
4	58 (35.2)	107 (64.8)	
pN stage			0.743
0	48 (42.1)	66 (57.9)	
1	27 (36.5)	47 (63.5)	
2	29 (40.3)	43 (59.7)	
TNM stage			0.283
I	12 (60.0)	8 (40.0)	
II	28 (36.4)	49 (63.6)	
III	48 (39.0)	75 (61.0)	
IV	16 (40.0)	24 (60.0)	
Historical type			1.000
Adenocarcinoma	98 (40.0)	147 (60.0)	
Mucinous adenocarcinoma	6 (40.0)	9 (60.0)	
Grade			0.499
Well-Moderately differentiated	77 (40.7)	112 (59.3)	
Moderately-Poor differentiated	19 (34.5)	36 (65.5)	
Unknown	8 (50.0)	8 (50.0)	
Vessel Invasion			0.408
Yes	39 (45.3)	47 (54.7)	
No	64 (37.6)	106 (62.4)	
Unknown	1 (25.0)	3 (75.0)	
Nerve Invasion			0.404
Yes	14 (34.1)	27 (65.9)	
No	90 (41.1)	129 (58.9)	

Bold emphasis indicates a statistically significant p-value

**Table 2:** Univariate and multivariate analysis of prognostic factors for disease-free survival in CRC patients

Variable	Univariate analysis		Multivariate analysis	
	HR (95% CI)	*P* value	HR (95% CI)	*P* value
UTX expression				
Low expression	Reference		Reference	
High expression	0.595 (0.383–0.925)	**0.021**	0.619 (0.394–0.972)	**0.037**
Age				
≤ 60	Reference		NA	
> 60	1.140 (0.763–1.703)		0.522	
Sex				
Male	Reference		NA	
Female	0.762 (0.503–1.155)		0.200	
Tumor Location				
Colon	Reference		NA	
Return	0.809 (0.544–1.202)		0.293	
Preoperative Serum CEA				
Normal	Reference		Reference	
Elevated	2.606 (1.731–3.922)	** < 0.001**	1.985 (1.302–3.024)	**0.001**
pT stage				
T2	Reference		Reference	
T3	2.621 (0.845–8.127)	0.095	2.542 (0.805–8.029)	0.112
T4	6.546 (2.398–17.872)	** < 0.001**	4.074 (1.466–11.321)	**0.007**
pN stage				
0	Reference		Reference	
1	3.157 (1.836–5.427)	** < 0.001**	0.777 (0.378–1.599)	0.494
2	4.434 (2.621–7.501)	** < 0.001**	0.932 (0.463–1.875)	0.843
TNM stage				
I + II	Reference		Reference	
III + IV	6.041 (3.298–11.067)	** < 0.001**	6.111 (2.469–15.122)	** < 0.001**
Histologic type				
Adenocarcinoma	Reference		NA	
Mucinous adenocarcinoma	0.870 (0.354–2.141)	0.762		
Grade				
Well-Moderately differentiated	Reference		NA	
Moderately-Poor differentiated	1.268 (0.512–3.141)	0.608		
Vessel invasion				
No	Reference		NA	
Yes	1.240 (0.171–8.976)	0.831		
Nerve invasion				
No	Reference		Reference	
Yes	1.767 (1.107–2.821)	**0.017**	1.123 (0.689–1.830)	0.642

Bold emphasis indicates a statistically significant p-value

**Table 3:** Univariate and multivariate analysis of prognostic factors for overall survival in CRC patients

Variable	Univariate analysis		Multivariate analysis	
	HR (95% CI)	*P* value	HR (95% CI)	*P* value
UTX expression				
Low expression	Reference		Reference	
High expression	0.615 (0.377–1.001)	0.051	0.683 (0.414–1.125)	0.134
Age				
≤ 60	Reference		NA	
> 60	1.233 (0.793–1.918)	0.353		
Sex				
Male	Reference		NA	
Female	1.233 (0.793–1.918)	0.308		
Tumor location				
Colon	Reference		NA	
Return	0.699 (0.450–1.086)	0.110		
Preoperative serum CEA				
Normal	Reference		Reference	
Elevated	2.364 (1.501–3.722)	** < 0.001**	1.689 (1.060–2.694)	**0.028**
**pT stage**				
T2	Reference			
T3	4.245 (0.930–19.377)	0.062	3.915 (0.840–18.251)	0.082
T4	10.531 (2.579–42.994)	**0.001**	6.871 (1.649–28.622)	**0.008**
pN stage				
0	Reference		Reference	
1	2.858 (1.580–5.172)	**0.001**	0.586 (0.280–1.227)	0.156
2	4.143 (2.334–7.353)	** < 0.001**	0.708 (0.346–1.451)	0.346
TNM stage				
I + II	Reference		Reference	
III + IV	6.493 (3.240–13.011)	** < 0.001**	8.739 (3.227–23.667	** < 0.001**
Histologic type				
Adenocarcinoma	Reference		NA	
Mucinous adenocarcinoma	0.857 (0.314–2.344)		0.764	
Grade				
Well-Moderately differentiated	Reference		NA	
Moderately-Poor differentiated	1.749 (0.548–5.583)		0.345	
Vessel invasion				
No	Reference		NA	
Yes	0.990 (0.136–7.201)	0.992		
Nerve invasion				
No	Reference		Reference	
Yes	1.573 (0.931–2.659)	0.091	0.938 (0.543–1.620)	0.818

Bold emphasis indicates a statistically significant p-value

### UTX depletion promotes CRC tumorigenesis in vivo

Next, we generated mice with specifically depleted *Utx* alleles in intestinal epithelial cells by crossing mice expressing *Villin*-Cre and Floxed *Utx* (*Utx*^f/f^ or *Utx*^f/y^) mice (Additional file 2: Fig. S1A). When we crossed *Utx*^−/+^ and *Utx*^−/y^ mice, among a total of 327 little mid-born expressing *Villin*-Cre, only mice with *Utx*^+/y^; *Villin*-Cre (n = 103), *Utx*^−/+^; *Villin*-Cre (n = 115) and *Utx*^−/y^; *Villin*-Cre (n = 109) genotypes were born. This could be due to the fact that the deletion of both copies of *Utx* in intestinal epithelial cells results in the embryonic lethality in female mice, while the homologous *Uty* gene may partially compensate for *Utx* loss in male mice. Western blotting confirmed the efficiency of *UTX* depletion in male mice’s large intestines (Additional file 2: Fig. S1B). Over a 16 months period, no abnormalities were detected in the appearance and structure of the large intestine in the *Utx*^−/y^ genotype mice, where *Utx* was depleted in the intestinal epithelial cells (Additional file 2: Fig. S1C and D).

**Fig. 2:**
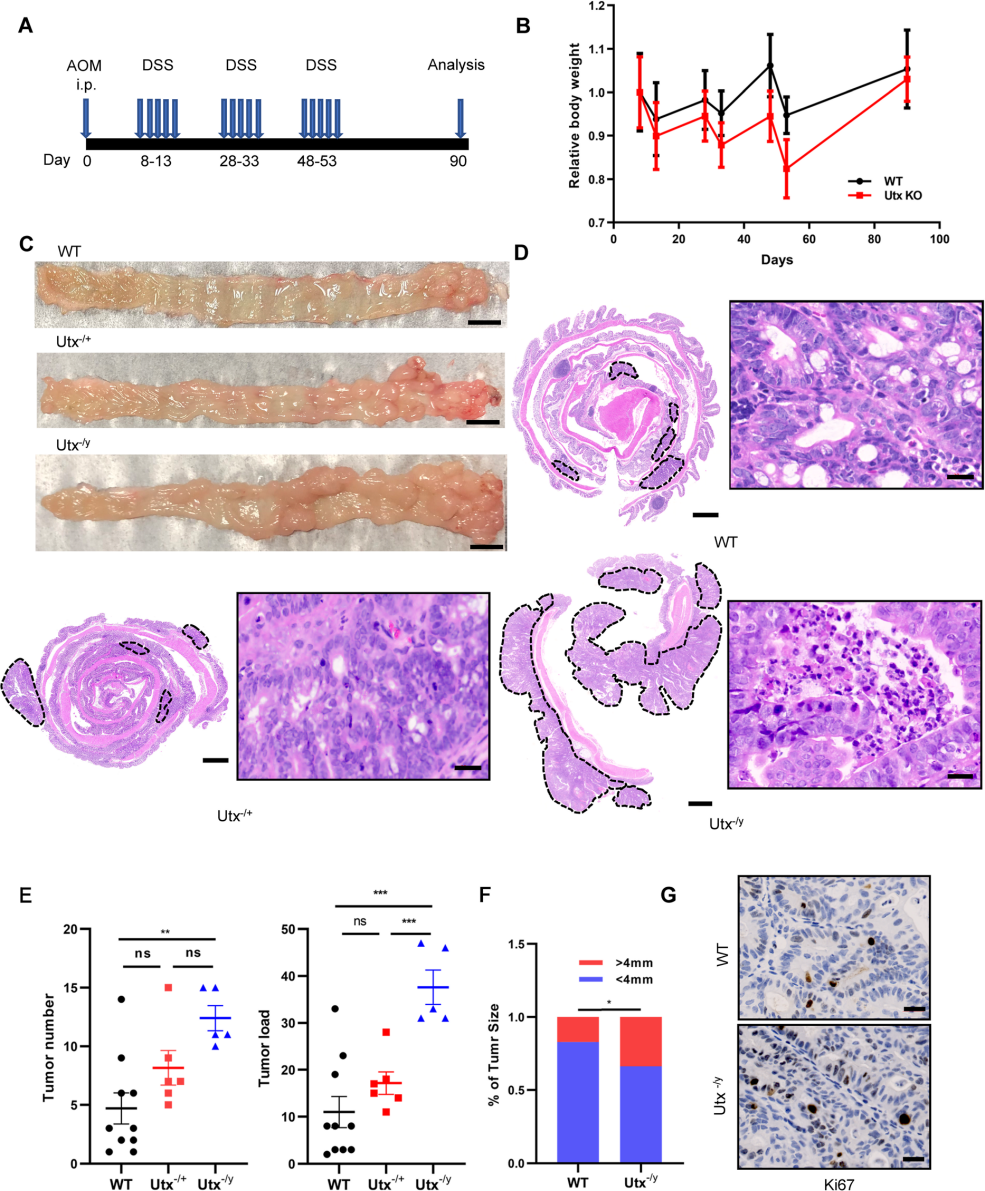
Conditional deletion of *Utx* in intestinal epithelial cells increases susceptibility to AOM/DSS-induced colorectal tumorigenesis. **A** Schematic model of the AOM/DSS protocol used to induce colorectal tumors in C57BL/6 mice. i.p., intraperitoneal. **B** Relative body weight of WT and *Utx*^−/x^/*Utx*^−/y^ was recorded during AOM/DSS treatment **C** and **D** Representative macroscopic (**C**) and H & E staining images (**D**) of large intestines from WT (n = 10), *Utx*^−/x^ (n = 6), and *Utx*^−/y^ (n = 5) mice at the end of the AOM/DSS protocol. Tumors were surrounded by dotted lines in representative images of H&E staining. Scale bar in (**C**), 5000 μm. Scale bar in (**D**), 1000 μm (left), and 20 μm (right), respectively. **E** and **F** Tumor number, tumor load (E), and size distribution of tumors (F) from large intestines of WT (n = 10), *Utx*^−/x^ (n = 6), and *Utx*^−/y^ (n = 5) mice. **G** Representative IHC images of Ki67 staining in large intestines of WT and *Utx*^−/y^ mice post AOM/DSS-induced CRC tumorigenesis. Scale bar, 20 μm. Data information: In (**B** and **E**), data are presented as mean ± SEM (one-way ANOVA with Dunnett’s multiple comparisons test). In (**F**), data are presented as rates (chi-square test). ns, non-significance, ^*^P < 0.05, ^**^P < 0.01, ^***^P < 0.001

Subsequently, AOM was combined with DSS to induce colitis and carcinoma (Fig. 2A). IHC was applied to determine the levels of UTX and H3K27me3. Consistent with a previous finding [15], *Utx* deletion led to increased H3K27me3 levels in intestinal tumors (Additional file 2: Fig. S1E).

During AOM/DSS treatment, four *Utx*^−/y^ mice and one *Utx*^−/+^ mouse while no deaths were observed in WT mice. As expected, the mice with different genotypes developed tumors located mainly in the distal to middle colorectum following AOM/DSS treatment. *Utx*^−/+^ and *Utx*^−/y^ mice showed marginal evidence of greater body weight loss than WT mice during AOM/DSS treatment (Fig. 2B), suggesting that the loss of *Utx* facilitates colitis and intestinal damage. We also observed that *Utx*^−/+^ and *Utx*^−/y^ mice had more macroscopic tumors and a higher average tumor load than WT mice (Fig. 2C–E). Moreover, *Utx*^−/y^ mice showed a more pronounced tumor load than *Utx*^−/+^ mice. Importantly, *Utx*^−/y^ mice formed larger tumors than WT mice (P = 0.049) (Fig. 2F). And the large intestines of *Utx*^−/y^ mice exhibited higher proliferative rates as compared with WT mice, as evidenced by the Ki67 signals (Fig. 2G). These results demonstrated that loss of *Utx* promoted tumorigenesis in the intestinal epithelial cells, and the tumor suppressive role of *Utx* is dose-dependent.

### UTX regulates CRC cell proliferation, in part, through governing EMP1 and AUTS2 expression

**Fig. 3:**
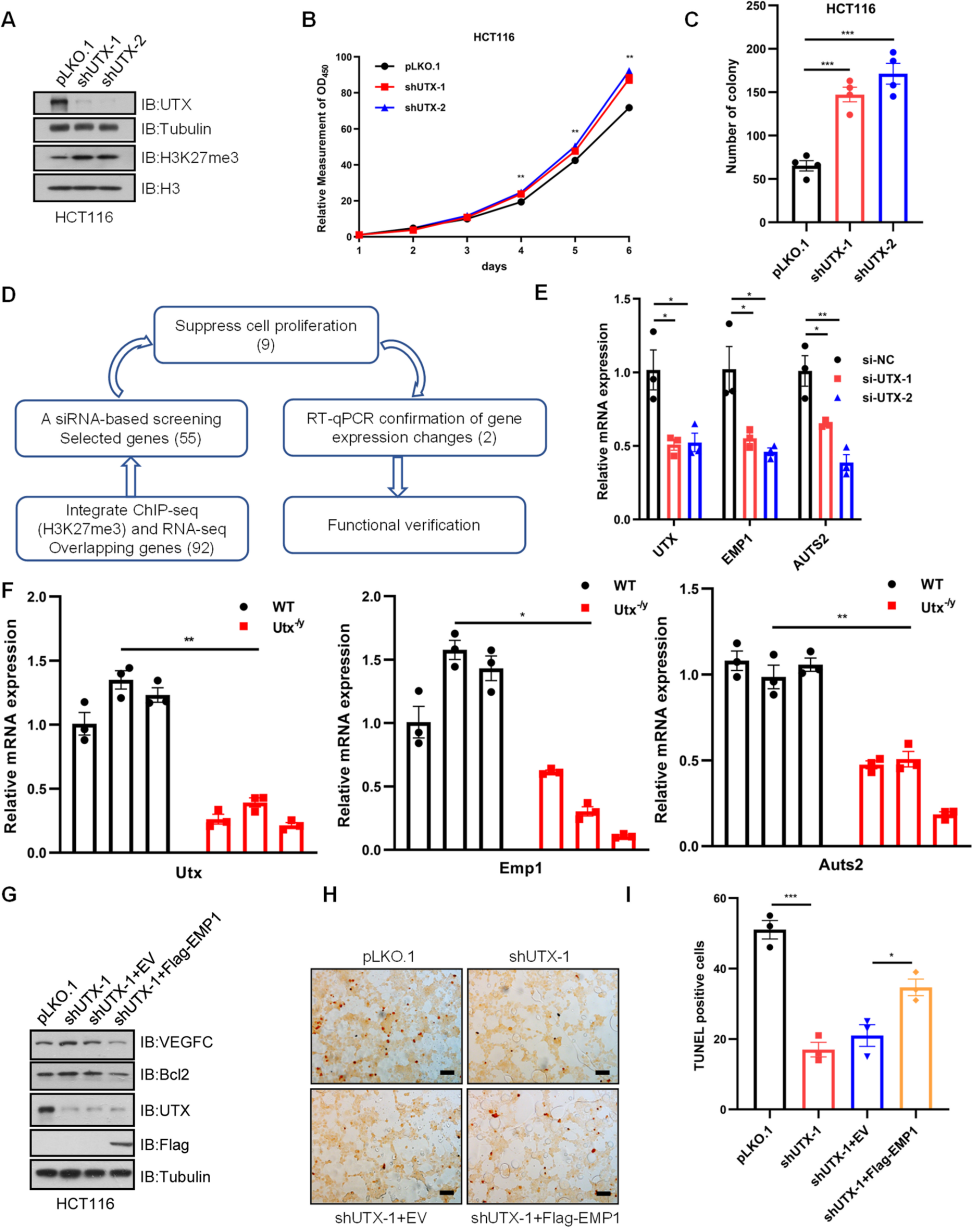
UTX inhibits tumorigenesis by upregulating the expression of EMP1 and AUTS2 in HCT116 cells. **A** IB of whole cell lysate (WCL) derived from HCT116 cells infected with lentiviruses expressing pLKO.1 or sh*UTX*. **B** Cell growth curve analysis of HCT116 cells infected with indicated lentiviruses. **C** Statistical analysis of soft agar assays in HCT116 cells infected with indicated lentiviruses. **D** Workflow for screening potential tumor suppressive target genes of UTX. **E** qPCR confirmation of *UTX*, *EMP1*, and *AUTS2* expression changes in HCT116 cells transfected with si*UTX* or negative control (NC). **F** qPCR detection of *Utx, Emp1*, and *Auts2* expression in intestinal epithelial cells from WT and *Utx*^−/y^ mice. **G** IB analysis of WCL from HCT116 cells infected with indicated lentiviruses.**H** Apoptosis of HCT116 cells infected with indicated lentiviruses was detected by TUNEL assay and observed under light microscopy. Scale bar, 50 μm. **I** Statistical analysis of TUNEL-positive cells in different groups. Data information: In (B, C, E, and F), data are represented as means ± SEM (two-tailed Student’s t-test). In (I), data are represented as means ± SEM (one-way ANOVA with Dunnett’s multiple comparisons test). ^*^P < 0.05, ^**^P < 0.01, ^***^P < 0.001

In order to investigate the mechanism of UTX function in CRC, *UTX*-knockdown HCT116 cells were generated using two independent shRNA constructs (Fig. 3A). As expected, the depletion of *UTX* facilitated the proliferation of HCT116 cells, as evidenced by Cell Counting Kit-8 (CCK8) assay and anchorage-independent soft agar assay (Fig. 3B and C). Since UTX is an epigenetic enzyme regulating H3K27me3, its function is expected to involve gene-specific H3K27me3 levels and subsequent changes in transcription. Thus, we performed ChIP-seq and transcriptome analysis of normal colorectal tissues from WT and *Utx* knockout mice (8-weeks-old, male) to elucidate how *UTX* deficiency drives an oncogenic program. This analysis identified 92 genes with > 1.5-fold increment of H3K27me3 modifications and > 33.3% decrease in transcripts upon *Utx* depletion. Among these genes, 55 genes were further selected for a siRNA-based screening in human CRC cell line HCT116 to identify conserved target genes mediating the growth inhibitory function of human UTX based on their gene function and literature evidence. Out of the 55 genes tested in the screening, the depletion of 9 genes (comprising *FRMPD1*, *HPSE2*, *EMP1*, *SELL*, *IFIT2*, *SLC8A1*, *SLC44A5*, *LIMCH1*, and *AUTS2*) significantly enhanced cell proliferation. Next, we employed qPCR to verify the expression changes in these 9 genes in mice intestinal tissues and HCT116 cells upon *UTX* depletion or knockdown, and we validated their function in suppressing HCT116 cell proliferation. Eventually, *AUTS2* and *EMP1* were identified as the significant UTX target genes as their expression showed dependence on *UTX* depletion in both mice tissue and HCT116 cells (Fig. 3D–F). The increased H3K27me3 modifications in *Emp1* and *Auts2* gene locus upon *Utx* depletion were displayed by genomic snapshots (Additional file 3: Fig. S2A). To investigate whether Utx directly regulates *Emp1* and *Auts2*, we performed chromatin immunoprecipitation followed by quantitative PCR (ChIP-qPCR). Consistently, *Utx* depletion caused significantly an increased H3K27me3 around the promoter of *Emp1* and *Auts2* (Additional file 3: Fig. S2B)*.* Typically, transcriptome sequencing revealed 401 significantly downregulated and 211 significantly upregulated genes upon *Utx* depletion (Additional file 3: Fig. S2C). Surprisingly, gene ontology (GO) analysis reflected that most biological processes affected were associated with immune processes (Additional file 3: Fig. S2D). Knockdown of *EMP1* or *AUTS2* significantly enhanced HCT116 cell proliferation in vitro (Fig. S3A-E). To further strengthen the potential functional role of EMP1 and AUTS2 as UTX’s major downstream genes to regulate cell growth, re-expression of EMP1 and AUTS2 was effectuated in human colorectal cancer cell line HCT116 post-sh*UTX* treatment. Interestingly, the overexpression of EMP1 or AUTS2 largely rescued the increased growth phenotype caused by *UTX* depletion in the anchorage-independent soft agar assay (Additional file 4: Fig. S3F and G). Additionally, we analyzed *UTX*, *EMP1*, and *AUTS2* mRNA expression in CRC patients derived from the TCGA database, and found a positive correlation between *UTX*-*EMP1* and *UTX*-*AUTS2* expression levels (Additional file 4: Fig. S3H). It is worth noting that AUTS2 is a component of the PRC1 complex, which regulates the development of neuronal gene expression [30]. Although several studies reported its possible function in lymphoma or leukemia, the mechanistic role of AUTS2 in cancer remains largely unclear.

EMP1 (Epithelial Membrane Protein1) has been implicated in various cancer types and is regarded to promote cancer progression by modulating cell growth and metastasis [31–34]. Unlike other cancer types, EMP1 has been implicated in CRC as a putative tumor suppressor, which may inhibit cell migration and promote apoptosis [35]. To explore the underlying mechanisms by which EMP1 inhibited HCT116 cell proliferation, the western blotting analysis demonstrated downregulation of *EMP1* upregulated VEGFC, Bcl-2 expression and downregulated Caspase-9 expression (Additional file 4: Fig S3A), consistent with the previous studies [35, 36]. Moreover, overexpression of EMP1 partially counteracted the suppressive role of UTX knockdown on apoptosis in HCT116 cells, as indicated by TUNEL assay and western blot (Fig. 3G–I). Taken together, these results suggested that EMP1 and AUTS2 play roles in mediating colorectal tumorigenesis driven by the loss of UTX.

### *Utx*-deficient CRC exhibits sensitivity to EZH2 inhibitor treatment

**Fig. 4:**
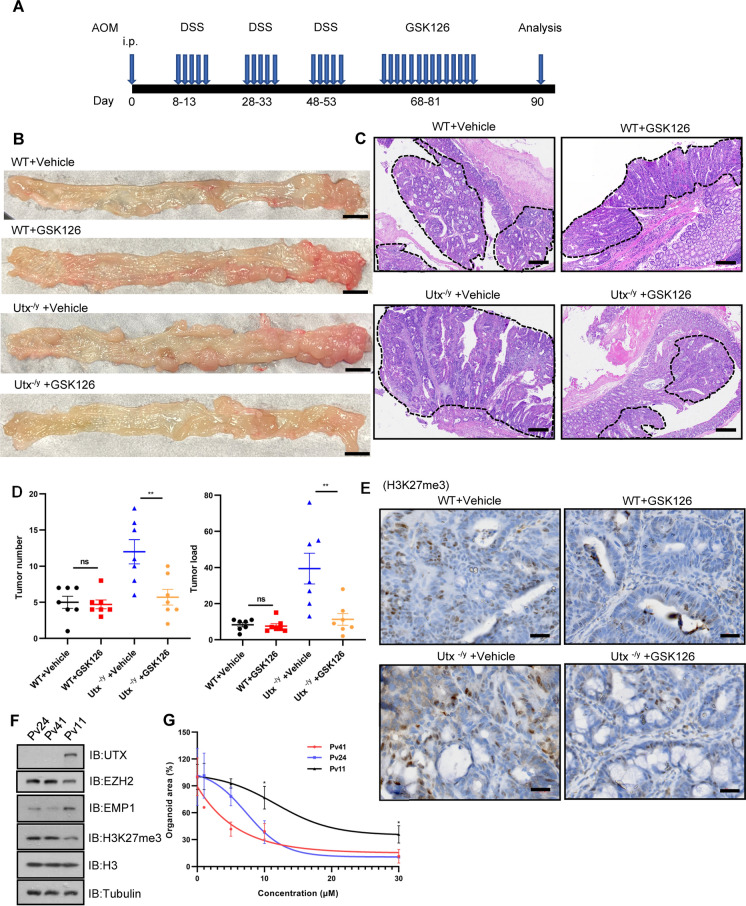
EZH2 inhibitor (GSK126) preferentially inhibits the growth of UTX-deficient colorectal tumors. **A** Schematic model of the protocol used to induce colorectal tumors in C57BL/6 mice and for the administration of the EZH2 inhibitor GSK126. i.p., intraperitoneal. **B** and **C** Representative macroscopic (**B**) and **H** & **E** staining images (**C**) of large intestines from WT and *Utx*^−/y^ mice treated with vehicle or GSK126 (n = 7, respectively). Tumors were surrounded by dotted lines in representative images of H&E staining. Scale bar in (**B**), 5000 μm. Scale bar in (**C**), 200 μm. **D** Tumor number and load from large intestines of experimental mice (n = 7/group, respectively). **E** Representative IHC staining of H3K27me3 expression in colorectal tumors from WT and *Utx*^−/y^ mice treated with vehicle or GSK126. Scale bar, 20 μm. **F** IB analysis of WCL from three human primary CRC organoids. **G** Dose–response curves of organoids treated with GSK126 (n = 3). Data information: In (**D** and **G**), data are presented as mean ± SEM (one-way ANOVA with Dunnett’s multiple comparisons test). ns, non-significance, ^*^P < 0.05, ^**^P < 0.01

Since the tumor suppressor role of UTX is related to H3K27me3 demethylation activity, tumors with low UTX activity may be driven by EZH2-related gene inhibition and are therefore sensitive to EZH2 inhibitors. Previous studies indicated that loss of UTX sensitizes the cells or tissues to EZH2 inhibition in multiple cancer settings [16, 18]. To explore whether UTX loss would sensitize CRC to EZH2 inhibition, spontaneous CRC was induced in WT and *Utx*^−/y^ mice through AOM/DSS treatment, followed by administration of EZH2 inhibitor GSK126 or vehicle control for additional two weeks (Fig. 4A). Compared to vehicle control, GSK126 treatment did not affect tumor number and tumor load in the WT mice. In contrast, treatment with GSK126 resulted in significant tumor regression in *Utx*^−/y^ mice (Fig. 4B–D) This observation is consistent with the finding that GSK126 treatment decreases the levels of H3K27me3 in both WT and *Utx*^−/y^ mice (Fig. 4E). To further explore the correlation between UTX expression and sensitivity to EZH2 inhibitor, three human CRC organoids were treated with different doses of GSK126 and their growth was examined. As indicated in Fig. 4F and G, organoid #Pv24 was the most sensitive organoid to GSK126 treatment with the lowest UTX expression, while organoid #Pv11 was the most resistant organoid to GSK126 treatment with the highest UTX expression, indicating an intrinsic reverse correlation between UTX expression and sensitivity to EZH2 inhibitor in human CRC. The expression of UTX target genes *EMP1* or *AUTS2* at protein and mRNA levels was increased post-GSK126 treatment (Additional file 4: Fig. S3I and J). These results suggested that UTX expression determines the sensitivity of CRC to EZH2 inhibitor, and low UTX expression may serve as a biomarker to identify CRC patients who can benefit from EZH2 inhibitor therapy.

### CRL4-COP1 E3 ligase complex ubiquitylates UTX for degradation

**Fig. 5:**
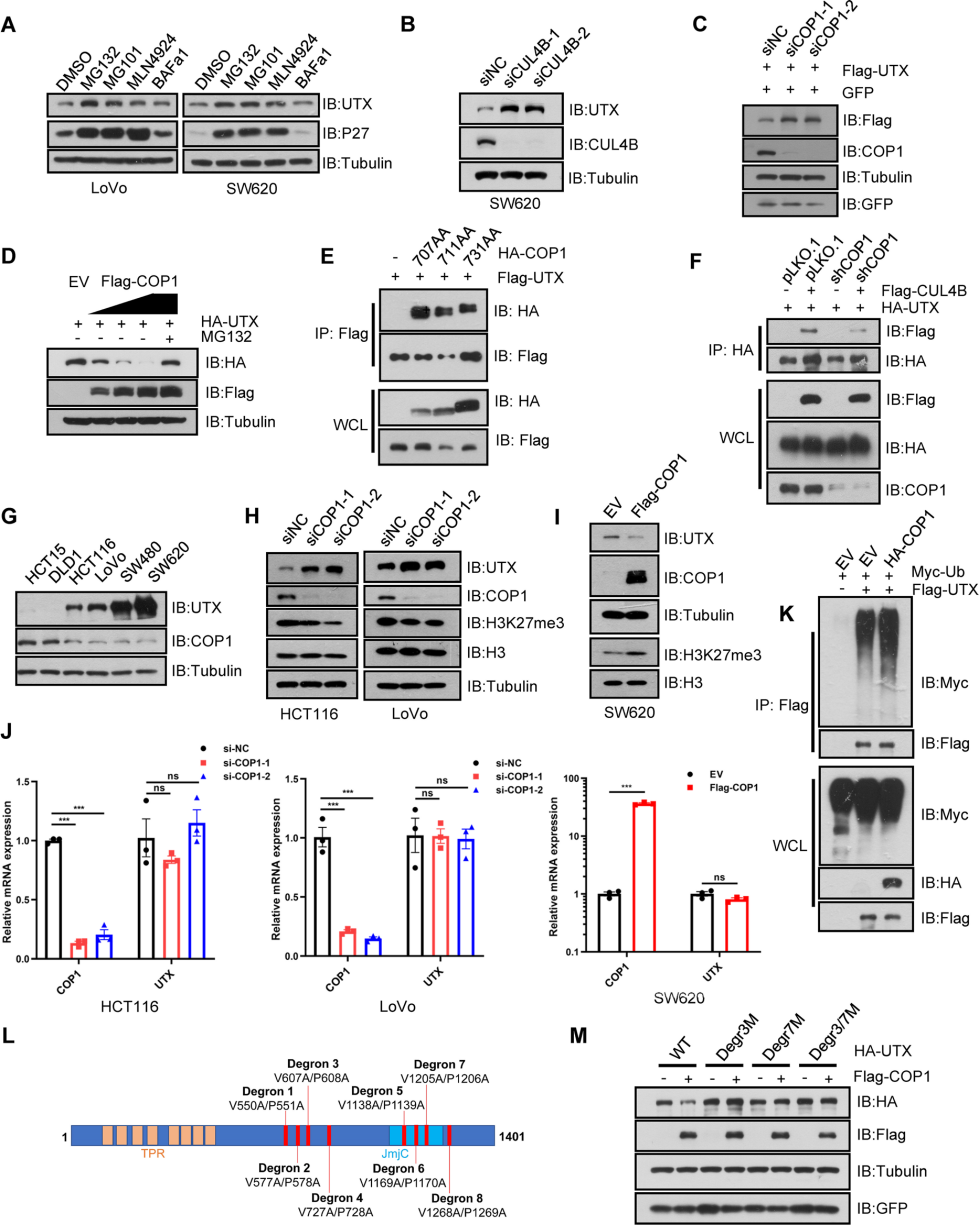
CRL4-COP1 E3 ligase complex ubiquitylates UTX for degradation. **A** IB analysis of WCL from LoVo and SW620 cells for indicated proteins. As indicated, cells were treated with DMSO, MG132, MG101, MLN4924, or Baf-A1 for 12 h before the cells were harvested. P27 was used as a positive control. **B** IB analysis of WCL from SW620 cells transfected with indicated siRNAs. **C** IB analysis of WCL from HEK293T cells co-transfected with Flag-UTX and si*COP1*. **D** IB analysis of WCL derived from HEK293T cells co-transfected with various doses of Flag-COP1 and HA-UTX constructs and treated with MG132 as indicated. **E** IB analysis of WCL and IP from HEK293T cells transfected with the indicated HA-COP1 splice variants and Flag-UTX constructs. Cells were treated with MG132 (20 µM) for 12 h before they were harvested. **F** IB analysis of WCL and IP from HEK293T cells transfected with the indicated shRNAs, empty vector (EV), Flag-CUL4B, and HA-UTX constructs. Cells were treated with MG132 (20 µM) for 12 h before they were harvested. **G** IB analysis of WCL from different colon cancer cell lines. **H** IB analysis of WCL from HCT116 and LoVo cells transfected with si*COP1* or NC. **I** IB analysis of WCL from SW620 cells infected with lentiviruses expressing EV or Flag-COP1. **J** qPCR analysis of *UTX* expression in indicated CRC cell lines transfected with si*COP1* or NC or Flag-COP1 or EV. **K** IB analysis of WCL and IP from HEK293T cells transfected with the indicated plasmids in the presence of MG132 (20 µM) for 12 h before they were harvested. **L** Illustration of the isoforms of UTX WT and different VP mutants. **M** IB analysis of WCL from HEK293T cells co-transfected with Flag-COP1 and WT or mutant HA-UTX. Data information: In (**J**), data are presented as mean ± SEM (two-tailed Student’s t-test). ns, non-significance, ^***^P < 0.001

Since the downregulation of UTX promoted CRC occurrence and progression, it is crucial to understand how UTX expression is regulated in CRC cells. In order to investigate the pathway governing UTX protein stability, we observed that treatment with the proteasome inhibitor MG132 or MG101 and the neddylation inhibitor MLN4924, but not lysosomal inhibitor BafA1, resulted in a marked increase in endogenous UTX abundance (Fig. 5A). This finding indicated that UTX is degraded via the ubiquitination-proteasome system, and the Cullin-RING E3 ligase (CRL) complexes are likely involved in mediating UTX ubiquitination, as MLN4924 inhibits CRL activity, which is well-documented. We then assessed how UTX interacts with and responds to various Cullin proteins. Although UTX interacted with CUL1 and CUL4B (Additional file 5: Fig. S4A), their expression levels were elevated only upon *CUL4B* knockdown (Additional file 5: Fig. S4B). Subsequently, we conducted a screening by transfecting siRNAs targeting all six Cullins in HCT116 cells and found that only *CUL4B* knockdown upregulated UTX protein levels (Additional file 5: Fig. S4C). Furthermore, we found that endogenous UTX protein levels were increased upon depletion of *CUL4B* in CRC cell line SW620 (Fig. 5B) Additionally, ectopic expression of CUL4B, DDB1 and DET1, the components of the CRL4 complex, reduced UTX expression in a dose-dependent manner (Additional file 5: Fig. S4D-F). These results strongly indicate that the CUL4B-based CRL4 complex is responsible for governing UTX protein stability.

Then, we tested siRNAs targeting several substrate recognition subunits of CUL4B E3 ligases (DDB2, COP1, DCAF7, and DCAF13) in HCT116 cells and found that *COP1* deficiency increased UTX protein levels (Additional file 5: Fig. S4G). Knockdown of *COP1* upregulated the ectopic UTX protein levels, while overexpression of COP1 decreased ectopic UTX protein levels (Fig. 5C and D). We also discovered that COP1 splice variants interacted with UTX (Fig. 5E). Furthermore, UTX interacted with each component of the complex, including COP1, DET1, Cul4B, DDB1 and RBX1 (Additional file 5: Fig. S4H). Consistently, ectopic expression of a mutation or deletion of the COP1 RING domain (C136A/C139A), which abolishes its intrinsic E3 ubiquitin ligase activity, decreased the abundance of UTX to the same extent (Additional file 5: Fig. S4I). Importantly, the depletion of endogenous *COP1* diminished the interaction between exogenous CUL4B and UTX (Fig. 5F), further confirming that COP1 is the substrate recognition subunit of the CRL4 complex that modulates UTX protein stability. Moreover, an inverse correlation was observed between UTX and COP1 proteins in CRC cell lines (Fig. 5G). Importantly, depletion of *COP1* in HCT116 and LoVo cells caused a distinct increase in endogenous UTX expression and reduced H3K27me3 expression (Fig. 5H). In contrast, ectopic expression of COP1 reduced the endogenous UTX expression in SW620, HCT116, and LoVo cells without affecting the transcription of *UTX* gene (Fig. 5I, J and Additional file 5: Fig S4J-M). Consistently, COP1 also promoted ubiquitination of UTX transfected in HEK293T cells (Fig. 5K).

Typically, COP1 interacts with “VP” motifs of its substrates, which are termed destruction “degron” sequences [25]. Thus, we mutated each of the eight conserved “VP” motifs (VP to AA) to identify the putative COP1-degron in UTX (Fig. 5L). Strikingly, only mutation in degron 3 (V607A/P608A) or degron 7 (V1205A/P1206A) impaired COP1-mediated UTX degradation (Fig. 5M and Additional file 6: Fig S5A). Taken together, these results indicate that the CRL4-COP1 complex governs UTX stability and H3K27me3 levels in CRC cells, with COP1 recognizing specific degron sequences in UTX to facilitate its degradation.

### COP1 promotes CRC partially via degrading UTX

**Fig. 6:**
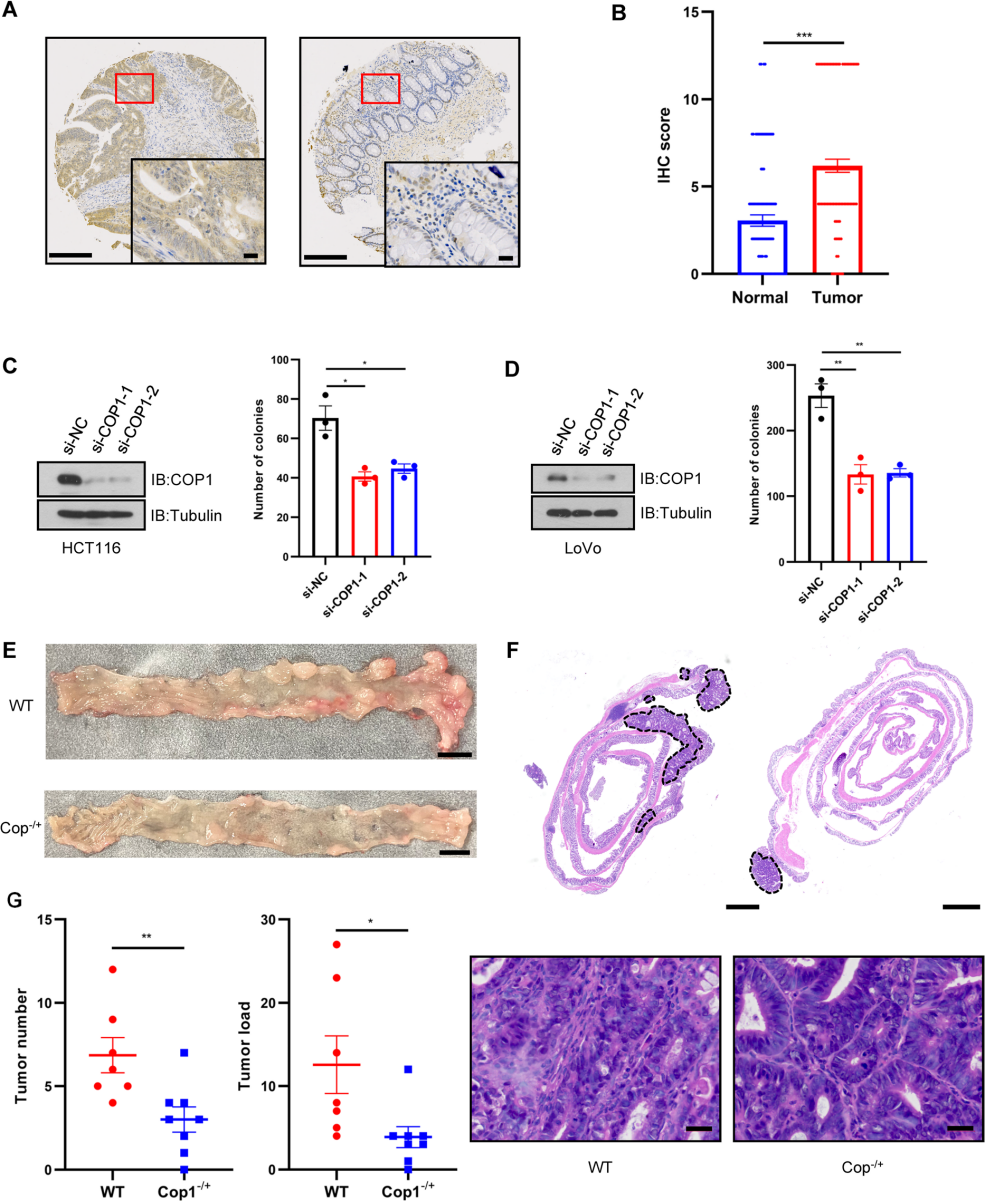
COP1 is an oncogenic protein in CRC. **A** Representative IHC images of COP1 staining in paired CRC and surrounding normal tissues. Scale bar, 100 μm (left) and 20 μm (right). **B** COP1 protein expression assessed by IHC scores of TMA in paired CRC and surrounding normal tissues (N = 104). **C** IB analyses of WCL (left) and statistical analysis of colony formation assays (right) in HCT116 cells treated with si*COP1* or NC. **D** IB analyses of WCL (left) and statistical analysis of colony formation assays (right) in LoVo cells treated with si*COP1* or NC. **E** and **F** Representative macroscopic (E) and H&E staining images (F) of large intestines from WT (n = 7) and *Cop1*^−/+^ (n = 8) mice at the end of the AOM/DSS protocol. Tumors were surrounded by dotted lines in representative images of H&E staining. Scale bar in (E), 5000 μm. Scale bar in (F), 1000 μm (upper), and 20 μm (bottom). **G** Tumor number and load from large intestines between WT and *Cop1*^−/+^ mice. Data information: In (B, C, D and G), data are presented as mean ± SEM (two-tailed Student’s t-test). ^*^P < 0.05, ^**^P < 0.01, ^***^P < 0.001

Since COP1 negatively regulates UTX expression in intestinal tissues, we speculated that COP1 might act as an oncoprotein in CRC. Analysis of TCGA, GTEx, and GEO databases revealed that *COP1* mRNA expression was upregulated in colon cancer or adenomas compared to normal colon tissues (Additional file 6: Fig. S5B and C). Immunoblotting of six pairs of CRC tumors and adjacent normal tissues confirmed that COP1 expression was upregulated in CRC tumor tissues (Additional file 6: Fig. S5D). Next, we detected the COP1 expression in paired CRC and normal (N = 104) specimens in TMA using IHC staining. The results demonstrated that COP1 expression was upregulated in CRC tissues compared to normal tissues (Fig. 6A and B). Moreover, siRNA-mediated *COP1* deficiency dramatically impaired the colony formation capacity of HCT116 and LoVo cells (Fig. 6C and D). Therefore, COP1 is a putative oncoprotein in CRC tumor cells. Next, we generated mice with specifically deleted *Cop1* alleles in intestinal epithelial cells by crossing flox *Cop1* (*Cop1*^f/f^) mice with *Villin*-Cre mice (Additional file 6: Fig. S5E). When we crossed *Villin*-Cre;*Cop1*^−/+^ and *Villin*-Cre;*Cop1*^−/+^ mice, *Villin*-Cre;*Cop1*^−/−^ mice were unavailable over the 2-year period, which could be ascribed to embryonic lethality due to the deletion of both copies of *Cop1* in intestinal epithelial cells. Next, we utilized WT and *Cop1*^−/+^ mice to generate a spontaneous CRC model following AOM/DSS treatment. Initially, eight male mice were used in each group, but one mouse in the WT group died during AOM/DSS treatment. Subsequently, we observed that *Cop1*^−/+^ mice displayed fewer macroscopic tumors and a low average tumor load than WT mice (Fig. 6E–G). These observations further support the notion of an oncogenic role of COP1 in spontaneous CRC, consistent with the findings of elevated COP1 levels in clinical CRC cases.

**Fig. 7:**
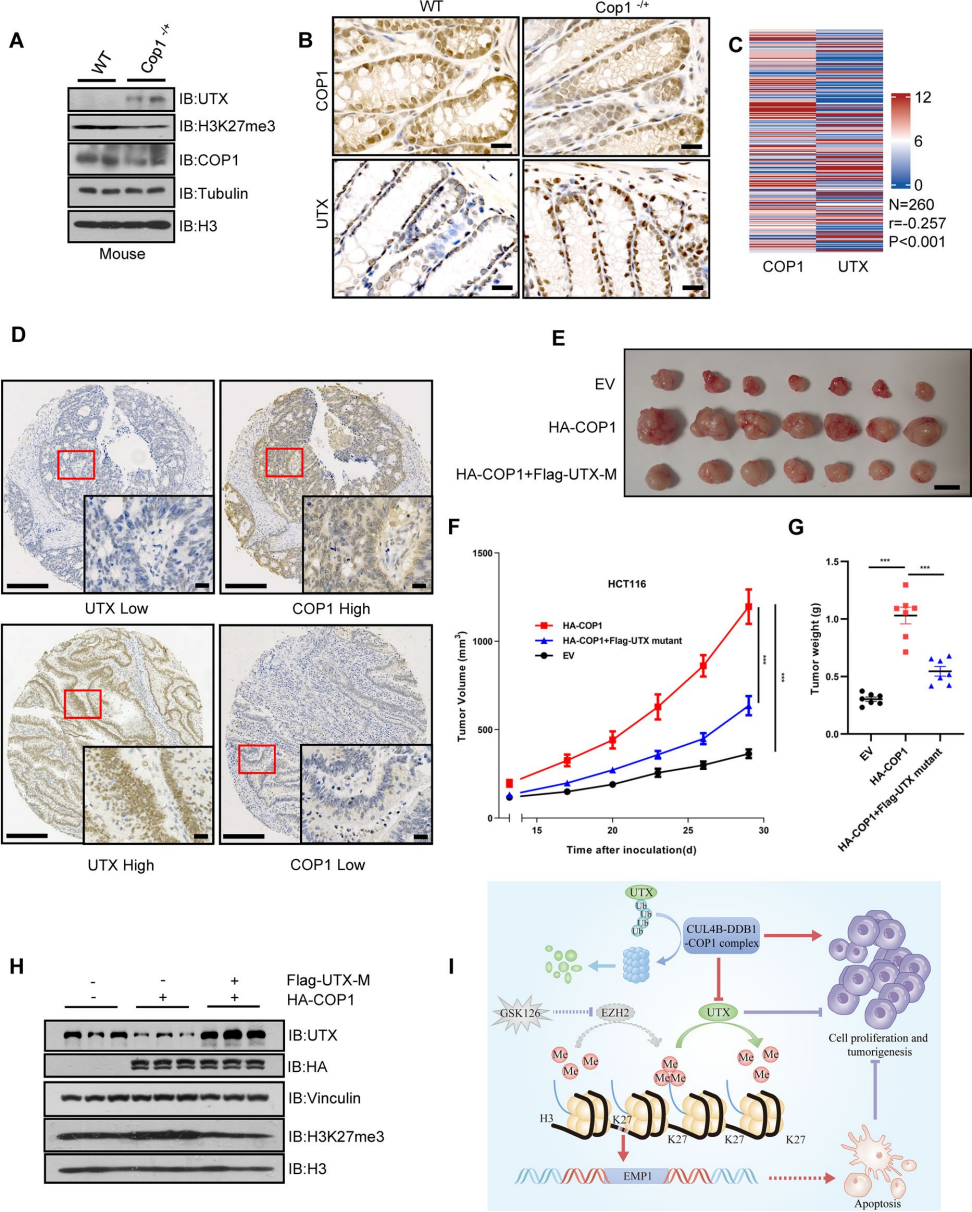
COP1 promotes CRC partially by degrading UTX. **A** and **B** IB analysis (**A**) and representative IHC staining (B) of the indicated proteins in intestinal epithelial cells of WT and *Cop1*^−/+^ mice. Scale bar, 20 μm. **C** Significant inverse correlation (r = -0.257, P < 0.001) between COP1 and UTX expression was determined by Pearson’s correlation analysis of CRC tissue microarray. **D** Representative IHC images of COP1 and UTX staining in CRC samples. Scale bar, 100 μm (left) and 20 μm (right). **E** and **F** Xenograft tumorigenesis of HCT116 cells with ectopic HA-COP1 or Flag-UTX-mutant (VP-AA) expression (n = 7, respectively). Scale bar, 1 cm. **G** Tumor weight of nude mice in different groups. **H** IB analysis of indicated proteins in tumors from different groups (n = 3). **I** Graphic model of dysregulated UTX contributing to CRC tumorigenesis. Data information: In (**F** and **G**), data are presented as mean ± SEM (one-way ANOVA with Dunnett’s multiple comparisons test). ^***^P < 0.001

To investigate the physiological role of COP1 in regulating UTX, we detected the protein expression of UTX in large intestines of WT and *Cop1*^−/+^ mice. Reduced COP1 expression increased the protein levels of UTX in mouse intestinal tissues, as detected by both immunoblotting and IHC staining (Fig. 7A and B). Then, we stained 260 CRC tumor tissues by the IHC method to determine the protein expression of COP1 and UTX. As indicated, the COP1 signal was high in most CRC tumors and significantly correlated with lower UTX expression (r = -0.257, P < 0.001, Fig. 7C and D). These findings suggest COP1 may facilitate CRC growth via UTX degradation. To investigate this hypothesis, we performed xenograft tumorigenesis experiment using HCT116 cells that ectopically expressed HA-COP1 and Flag-UTX mutant (V607A/P608A/V1205A/P1206A) resistant to COP1-mediated degradation (Additional file 6: Fig. S5F). The ectopic expression of COP1 dramatically promoted tumorigenesis compared to parental HCT116 cells, while expression of non-degradable UTX essentially reversed the tumor-promoting effect derived from COP1 overexpression (Fig. 7E–G). Importantly, this reversal was associated with increased H3K27me3 levels upon COP1 overexpression and reduced H3K27me3 levels after overexpression of non-degradable UTX in the tumors from xenograft experiment (Fig. 7H). These results suggested that COP1-mediated UTX degradation is crucial for COP1-driven CRC tumorigenesis.

## Discussion

The oncogenic function and regulation of EZH2, the major H3K27 methyltransferase, in CRC have been well-documented [37–40]. Conversely, the functional and regulatory mechanism of the H3K27me3 histone demethylase UTX in CRC is yet unclear. In the present study, we found that UTX protein levels were downregulated in advanced stages of CRC as examined by immunostaining. Moreover, low UTX expression was associated with poor patient survival, indicating a putative tumor suppressor role of UTX in human CRC (Fig. 1). Next, we observed that UTX loss significantly increased tumor numbers and burden in the AOM/DSS-induced CRC model. These results suggested that UTX functions as a tumor suppressor in CRC, and reduced UTX may drive the progression of the disease. Since reduced UTX expression in intestinal tissue significantly increased the H3K27me3 levels, which are enriched in CRC with more advanced disease stages, we speculated that targeting H3K27me3 may be an effective way to treat UTX loss-related CRC. Typically, spontaneous colorectal tumors derived from UTX-deficient intestines or human CRC organoids with low UTX expression exhibited sensitivity to EZH2 inhibitors, consistent with previous reports in other cancer types [16, 18]. Therefore, the EZH2 inhibitor could be a promising therapeutic option for CRC patients with UTX deficiency.

The combined analysis of *UTX*-depleted mice intestine and human CRC cells identified *EMP1* and *AUTS2* as the target genes modulated by UTX since their expression decreased upon *UTX* depletion and increased upon treatment with the EZH2 inhibitor GSK126. Notably, the depletion of *EMP1* and *AUTS2* promoted CRC cell proliferation, a phenotype similar to *UTX* knockdown. Reportedly, EMP1 has a possible cross-talk with the EGFR signaling pathway [41] and negatively regulates cell growth and metastasis in CRC [35], and its downregulation is observed in multiple cancers [36, 42]. In contrast, AUTS2 is frequently disrupted in patients with neurological disorders [30] but is less studied in cancer. Here, we characterized UTX as the epigenetic regulator of *EMP1* and *AUTS2* expression. Furthermore, the increased protein level of VEGFC and BCL2 as well as reduced cell apoptosis caused by depletion of *UTX* could be rescued by ectopic expression of *EMP1*, suggesting that *EMP1* is a significant effector gene mediating the tumor-suppressing function of UTX in CRC. However, during our screening process, we only focused on putative target genes mutually modulated by UTX in human CRC cells and mice intestines, particularly those that affect cell growth. Other putative UTX target genes (*FRMPD1*, *HPSE2*, *SELL*, *IFIT2*, *SLC8A1*, *SLC44A5*, and *LIMCH1*) were identified in ChIP-seq and RNA-seq analyses. These genes may also modulate CRC via various mechanisms other than regulating cell growth. Some specific human UTX target genes that mediate its tumor suppressor function in CRC might not have been discovered in the mouse model.

According to the COSMIC (the Catalogue of Somatic Mutations in Cancer) database and previous reports, genomic deletion or mutation of UTX is not a frequent event in CRC. Therefore, it is essential to understand how UTX expression is impaired in CRC. The current study indicated that the CUL4B-DDB1-COP1 E3 ligase complex governs UTX protein stability. Depletion of *COP1* in human CRC cells or mice intestinal tissue caused a marked increase in UTX expression and restricted tumorigenesis. The expression of COP1 was inversely correlated with UTX expression in human CRC specimens. Furthermore, we observed a remarkably enhanced growth of tumors upon ectopic expression of COP1 in the in vivo tumorigenesis assays. Importantly, ectopic expression of a non-degradable UTX mutant reversed the tumor-promoting effect of COP1 overexpression. Mechanistically, we have characterized that UTX is a substrate for the CUL4B-DDB1-COP1 E3 complex, but not for the COP1 intrinsic RING motif. We also identified two putative “VP” degron motifs in UTX that mediate COP1 recognition. Thus, the current study revealed the oncogenic role of COP1 in CRC, at least partially, by degrading UTX and inducing epigenetic changes. Additionally, it uncovers a critical layer of UTX regulation in CRC.

## Conclusions

The current study has provided insights into the tumor suppressor function of UTX in CRC. We identified its downstream effector genes, *EMP1* and *AUTS2,* which play crucial roles in mediating its tumor-suppressing effects. Additionally, we observed the downregulation of UTX caused by the CRL4-COP1 complex in CRC (Fig. 7I). Thus, our findings would shed new light on the epigenetic reprogramming and novel therapeutic option of CRC with UTX deficiency.

### Supplementary Information

**Additional file 1: Table S1.** Primer sequences for genotyping and qPCR. **Table S2.** siRNA sequences.

**Additional file 2: Figure S1.** Loss of UTX contributes to CRC. A Genotyping of *Utx* and *Villin*-CRE mice by PCR. B IB analysis of UTX expression in large intestines of WT and *Utx*^-/y^ mice. C and D Macroscopic image (C) and H&E staining (D) of large intestines derived from 16-month-old WT and *Utx*^-/y^ mice. Scale bar in (C), 5000 μm. Scale bar in (D), 1000 μm (upper) and 20 μm (bottom). E Representative IHC images of UTX and H3K27me3 staining in large intestines of WT and *Utx*^-/y^ mice post AOM/DSS-induced CRC tumorigenesis. Scale bar, 20 μm.

**Additional file 3: Figure S2.** *EMP1* and *AUTS2* are putative UTX target genes. **A** ChIP-seq tracks for H3K27me3 at *Emp1* and *Auts2* gene locus.** B **ChIP-qPCR of H3K27me3 modifications on the promoter regions of the indicated genes.** C** Volcano plots showing the differentially expressed genes in large intestines between WT and *Utx*^-/y^ mice.** D** GO enrichment analysis showed biological processes regulated by *Utx*. Data information: In (B), data are presented as mean±SEM (two-tailed Student’s t-test). ^*^P<0.05, ^**^P<0.01

**Additional file 4: Figure S3 **Functional correlation between *UTX* and *EMP1* or *AUTS2*.** A** IB analysis of WCL from HCT116 cells transfected with si*EMP1* or NC. **B** Cell growth curve analysis of HCT116 cells transfected with si*EMP1* or NC. **C** Proliferation of HCT116 cells transfected with indicated siRNA was detected by EdU assay and observed under light microscopy (left). Scale bar, 50 μm. Statistical analysis of EdU-positive cells in different groups (right). **D** IB analysis of WCL from HCT116 cells transfected with si*AUTS2* or NC. **E** Cell growth curve analysis of HCT116 cells transfected with si*AUTS2* or NC. **F **IB analysis of WCL from HCT116 cells infected with indicated lentiviruses. **G** Growth of HCT116 cells infected with indicated lentiviruses was detected by the anchorage-independent soft agar assay. Statistical analysis of soft agar assays in different groups. **H** *UTX* expression was positively correlated with *EMP1* and *AUTS2* in the TCGA dataset. **I** IB analysis of HCT116 cells treated with different concentrations of GSK126 (0 μM, 1 μM, 10 μM). **J** qPCR analysis of *EMP1* and *AUTS2* expression in HCT116 cells treated with vehicle or GSK126 (10 μM). Data information: In (B, C, E and J), data are presented as mean±SEM (two-tailed Student’s t-test). In (G), data are presented as mean±SEM (one-way ANOVA with Dunnett’s multiple comparisons test). ^*^P<0.05, ^**^P<0.01, ^***^P<0.001.

**Additional file 5: Figure S4. **CRL4-COP1 E3 complex modulates UTX stability. **A** IB analysis of WCL and IP from HEK293T cells co-transfected with HA-UTX and different Cullin family constructs. Cells were treated with MG132 (20 µM) for 12 h before they were harvested. **B** IB analysis of WCL from HEK293T cells co-transfected with Flag-UTX and sh*CUL1* or sh*CUL4B*. **C** IB analysis of WCL from HCT116 cells transfected with indicated siRNA targeting different Cullins. **D** IB analysis of WCL derived from HEK293T cells co-transfected with various doses of Flag-CUL4B together with HA-UTX constructs. Cells were treated with or without MG132, as indicated before they were harvested. **E** IB analysis of WCL derived from HEK293T cells co-transfected with various doses of Flag-DDB1 and HA-UTX constructs. Cells were treated with or without MG132, as indicated before they were harvested. **F** IB analysis of WCL derived from HEK293T cells co-transfected with various doses of Flag-DET1 and HA-UTX constructs. Cells were treated with or without MG132, as indicated before they were harvested.** G** IB analysis of WCL from HCT116 cells transfected with indicated siRNA targeting different CUL4B adaptor E3 ligases.** H** IB analysis of WCL and IP from HEK293T cells co-transfected with HA-UTX and CUL4B complex. Cells were treated with MG132 (20 µM) for 12 h before they were harvested.** I** IB analysis of WCL derived from HEK293T cells co-transfected with HA-UTX and Flag-COP1 (WT or C136A/C139A or △Ring). **J and K** IB (H) and qPCR analysis (I) of UTX expression in HCT116 cells infected with lentiviruses expressing EV or Flag-COP1. **L and M** IB (J) and qPCR analysis (K) of UTX expression in LoVo cells infected with lentiviruses expressing EV or Flag-COP1. Data information: In (K and M), data are presented as mean±SEM (two-tailed Student’s t-test). ns, non-significance, ^***^P<0.001

**Additional file 6: Figure S5.** COP1 is an oncogenic protein in CRC. A IB analysis of WCL from HEK293T cells co-transfected with Flag-COP1 and WT or mutant HA-UTX. B *COP1* mRNA levels in colon cancer tissues (n=471) and normal tissues (n=349) were determined from TCGA and GETx databases. C *COP1* mRNA levels in colorectal adenoma (n=32) and normal tissues (n=32) were determined from GDS2947. D IB analyses of COP1 expression in six pairs of random CRC samples. T, matched tumor tissues; N, adjacent normal specimens. E Genotyping of* Cop1*^f/-^ mice by PCR. F IB analysis of WCL from HCT116 cells with ectopic HA-COP1 or Flag-UTX-mutant (V607A/P608A/V1205A/P1206A) expression. Data information: In (B and C), data are presented as mean±SEM (two-tailed Student’s t-test), ^***^P<0.001

## Data Availability

RNA-seq data were deposited in the NCBI’s SRA (Accession: PRJNA734666, http://www.ncbi.nlm.nih.gov/sra?term=PRJNA734666). ChIP-seq data were deposited in the NCBI’s SRA (Accession: PRJNA752794, http://www.ncbi.nlm.nih.gov/sra?term=PRJNA752794). All data supporting the findings are available from the corresponding author upon reasonable request.

## Abbreviations

CRC: Colorectal cancer
UTX: The ubiquitously transcribed tetratricopeptide repeat on the X chromosome
CRL: Cullin-RING Ligase
COP1: Constitutive photomorphogenic 1
TMA: Tissue microarray
WT: Wild type
FUSCC: Fudan University Shanghai Cancer Center
TNM: Tumor-node-metastasis
AOM: Azoxymethane
IP: Immunoprecipitation
DSS: Dextran sodium sulfate
TCGA: The Cancer Genome Atlas
GTEx: Genotype-tissue expression
GEO: Gene Expression Omnibus
IHC: Immunohistochemistry
OS: Overall survival
DFS: Disease-free survival
