# Supplementary material for: CUL4B-DDB1-COP1-mediated UTX downregulation promotes colorectal cancer progression
Source: Exp Hematol Oncol. 2023 Sep 7;12:77. doi: 10.1186/s40164-023-00440-z (PMC10483726; doi:10.1186/s40164-023-00440-z)
Supplement: Supplementary file 1 — Additional file 1: Table S1. Primer sequences for genotyping and qPCR. Table S2. siRNA sequences. [file 40164_2023_440_MOESM1_ESM.docx]

**Supplementary Figure Legends**

**Figure S1.** **Loss of UTX contributes to CRC.** **A** Genotyping of *Utx* and *Villin*-CRE mice by PCR. **B** IB analysis of UTX expression in large intestines of WT and *Utx*^-/y^ mice. **C and D** Macroscopic image (C) and H&E staining (D) of large intestines derived from 16-month-old WT and *Utx*^-/y^ mice. Scale bar in (C), 5000 μm. Scale bar in (D), 1000 μm (upper) and 20 μm (bottom). **E** Representative IHC images of UTX and H3K27me3 staining in large intestines of WT and *Utx*^-/y^ mice post AOM/DSS-induced CRC tumorigenesis. Scale bar, 20 μm.

**Figure S2. *EMP1* and *AUTS2* are putative UTX target genes. A** ChIP-seq tracks for H3K27me3 at *Emp1* and *Auts2* gene locus. **B** ChIP-qPCR of H3K27me3 modifications on the promoter regions of the indicated genes. **C** Volcano plots showing the differentially expressed genes in large intestines between WT and *Utx*^-/y^ mice. **D** GO enrichment analysis showed biological processes regulated by *Utx*. Data information: In (B), data are presented as mean±SEM (two-tailed Student’s t-test). ^*^P<0.05, ^**^P<0.01.

**Figure S3** Functional correlation between *UTX* and *EMP1* or *AUTS2*. **A** IB analysis of WCL from HCT116 cells transfected with si*EMP1* or NC. **B** Cell growth curve analysis of HCT116 cells transfected with si*EMP1* or NC. **C** Proliferation of HCT116 cells transfected with indicated siRNA was detected by EdU assay and observed under light microscopy (left). Scale bar, 50 μm. Statistical analysis of EdU-positive cells in different groups (right). **D** IB analysis of WCL from HCT116 cells transfected with si*AUTS2* or NC. **E** Cell growth curve analysis of HCT116 cells transfected with si*AUTS2* or NC. **F** IB analysis of WCL from HCT116 cells infected with indicated lentiviruses. **G** Growth of HCT116 cells infected with indicated lentiviruses was detected by the anchorage-independent soft agar assay. Statistical analysis of soft agar assays in different groups. **H** *UTX* expression was positively correlated with *EMP1* and *AUTS2* in the TCGA dataset. **I** IB analysis of HCT116 cells treated with different concentrations of GSK126 (0 μM, 1 μM, 10 μM). **J** qPCR analysis of *EMP1* and *AUTS2* expression in HCT116 cells treated with vehicle or GSK126 (10 μM). Data information: In (B, C, E and J), data are presented as mean±SEM (two-tailed Student’s t-test). In (G), data are presented as mean±SEM (one-way ANOVA with Dunnett’s multiple comparisons test). ^*^P<0.05, ^**^P<0.01, ^***^P<0.001.

**Figure S4.** CRL4-COP1 E3 complex modulates UTX stability. **A** IB analysis of WCL and IP from HEK293T cells co-transfected with HA-UTX and different Cullin family constructs. Cells were treated with MG132 (20 µM) for 12 h before they were harvested. **B** IB analysis of WCL from HEK293T cells co-transfected with Flag-UTX and sh*CUL1* or sh*CUL4B*. **C** IB analysis of WCL from HCT116 cells transfected with indicated siRNA targeting different Cullins. **D** IB analysis of WCL derived from HEK293T cells co-transfected with various doses of Flag-CUL4B together with HA-UTX constructs. Cells were treated with or without MG132, as indicated before they were harvested. **E** IB analysis of WCL derived from HEK293T cells co-transfected with various doses of Flag-DDB1 and HA-UTX constructs. Cells were treated with or without MG132, as indicated before they were harvested. **F** IB analysis of WCL derived from HEK293T cells co-transfected with various doses of Flag-DET1 and HA-UTX constructs. Cells were treated with or without MG132, as indicated before they were harvested. **G** IB analysis of WCL from HCT116 cells transfected with indicated siRNA targeting different CUL4B adaptor E3 ligases. **H** IB analysis of WCL and IP from HEK293T cells co-transfected with HA-UTX and CUL4B complex. Cells were treated with MG132 (20 µM) for 12 h before they were harvested. **I** IB analysis of WCL derived from HEK293T cells co-transfected with HA-UTX and Flag-COP1 (WT or C136A/C139A or △Ring). **J and K** IB (H) and qPCR analysis (I) of UTX expression in HCT116 cells infected with lentiviruses expressing EV or Flag-COP1. **L and M** IB (J) and qPCR analysis (K) of UTX expression in LoVo cells infected with lentiviruses expressing EV or Flag-COP1. Data information: In (K and M), data are presented as mean±SEM (two-tailed Student’s t-test). ns, non-significance, ^***^P<0.001.

**Figure S5.** COP1 is an oncogenic protein in CRC. **A** IB analysis of WCL from HEK293T cells co-transfected with Flag-COP1 and WT or mutant HA-UTX.

**B** *COP1* mRNA levels in colon cancer tissues (n=471) and normal tissues (n=349) were determined from TCGA and GETx databases. **C** *COP1* mRNA levels in colorectal adenoma (n=32) and normal tissues (n=32) were determined from GDS2947. **D** IB analyses of COP1 expression in six pairs of random CRC samples. T, matched tumor tissues; N, adjacent normal specimens. **E** Genotyping of *Cop1*^f/-^ mice by PCR. **F** IB analysis of WCL from HCT116 cells with ectopic HA-COP1 or Flag-UTX-mutant (V607A/P608A/V1205A/P1206A) expression. Data information: In (B and C), data are presented as mean±SEM (two-tailed Student’s t-test), ^***^P<0.001.

**Supplementary Table 1. Primer sequences for genotyping and qPCR.**

| Gene | Primer Sequences |
| --- | --- |
| **Genotyping** |  |
| D367--Utx-CF | TGGCAGGCATTAAACCTGAATC |
| D368-Utx-CR | ACATAGCCCATGAATGCTCTGC |
| D369-Utx-CR | TAGCCAGTACGGTATTAGGTG |
| Cop1-L-LOXP | F: GCTTGTTTGAACACTGTATGT; R: GTAAAATGAAATGTGAATGGG |
| pVillin-Cre  **qPCR** | F: ATCAACGTTTTCTTTTCGG; R: ATTTGCCTGCATTACCGGTC |
| Utx | F: GCAAGTGCAGATACATGGTG; R: CCGTGGTCCAATTGTACAGC |
| Emp1 | F: TTGGTGCTACTGGCTGGTCT; R: AGCATCTTCATTGCCGTAGGA |
| Auts2 | F: GGAGGTCTCGATCACAGCG; R: TTCGGCTGAGGTGGACTCT |
| β-actin-mus | F: GGCTGTATTCCCCTCCATCG; R: CCAGTTGGTAACAATGCCATGT |
| hCOP1 | F: ACGACCTTTAGCCACATTGT; R: TAACTCCAGCAATCGCAAAA |
| hUTX | F: GACATTGAGGGAAGCTCTCA; R: ACTTGCATCAGGTCCTCCAT |
| hEMP1 | F: GTGCTGGCTGTGCATTCTTG; R: CCGTGGTGATACTGCGTTCC |
| hAUTS2 | F: ACGAGAAGCACTTACCAATGG; R: CGATCTGAGCTGTAGTGGTGT |
| hGAPDH | F: GTCATCAATGGAAATCCCATCA; R: CCAGTGGACTCCACGACGTAC |
| **ChIP-qPCR Primer** |  |
| Emp1 | F: TGCCCTACGTCCTTGACAAC; R: GCTGGGAGTGAGCCATCAAT |
| Auts2 | F: TCGCAGCGCTTCTTCCTCAG; R: ATTATAAAGGGGGAGGGGGTGA |

**Supplementary Table 2. siRNA sequences.**

| Gene | Sequences |
| --- | --- |
| hs-COP1-si-1 | F: GAAGAAGCAUACAUGACAAdTdT; R: UUGUCAUGUAUGCUUCUUCdTdT |
| hs-COP1-si-2 | F: AGUUACUAGUGCAGAAGAAdTdT; R: UUCUUCUGCACUAGUAACUdTdT |
| hs-UTX-si-1 | F: GACUAUGAGUCUAGUUUAAdTdT; R: UUAAACUAGACUCAUAGUCdTdT |
| hs-UTX-si-2 | F: GGUGGAAGCUAACAAUGAAdTdT; R: UUCAUUGUUAGCUUCCACCdTdT |
| hs-CUL4B-si-1 | F: GUUUUUGAGGCCUUCUAUAdTdT; R: UAUAGAAGGCCUCAAAAACdTdT |
| hs-CUL4B-si-2 | F: CAGUAAAGCCUGCUGAUCUdTdT; R: AGAUCAGCAGGCUUUACUGdTdT |
| hs-EMP1-si-1 | F: CCACAUCGCUACUGUUAUUdTdT; R: AAUAACAGUAGCGAUGUGGdTdT |
| hs-EMP1-si-2 | F: UACGGUAGAUGCAUCAGUAdTdT; R: UACUGAUGCAUCUACCGUAdTdT |
| hs-AUTS2-si-1 | F: CGAGACUCAUCUGUUAGUAdTdT; R: UACUAACAGAUGAGUCUCGdTdT |
| hs-AUTS2-si-2 | F: GCCUACAACAGCAGUAGCUdTdT; R: AGCUACUGCUGUUGUAGGCdTdT |
| hs-CUL1-si-1 | F: GGAUGAGAGUGUACUGAAAdTdT; R: UUUCAGUACACUCUCAUCCdTdT |
| hs-CUL1-si-2 | F: CCAGAUAUAUGGAGCUCUAdTdT; R: UAGAGCUCCAUAUAUCUGGdTdT |
| hs-CUL2-si-1 | F: GGCAAAUAUGUACGUCUUAdTdT; R: UAAGACGUACAUAUUUGCCdTdT |
| hs-CUL2-si-2 | F: GAAGAACAAGUACUUGUUAdTdT; R: UAACAAGUACUUGUUCUUCdTdT |
| hs-CUL3-si-1 | F: AAGGUGCGAGAAGAUGUATTdTdT; R: AAUACAUCUUCUCGCACCUUdTdT |
| hs-CUL3-si-2 | F: AACAACUUUCUUCAAACGCUAdTdT; R: UAGCGUUUGAAGAAAGUUGUUdTdT |
| hs-CUL4A-si-1 | F: GGAAGAGACUAAUUGCUUAdTdT; R: UAAGCAAUUAGUCUCUUCCdTdT |
| hs-CUL4A-si-2 | F: GAACAGCGAUCGUAAUCAAdTdT; R: UUGAUUACGAUCGCUGUUCdTdT |
| hs-CUL5-si-1 | F: GGCUAAUAGAGCACAAAUAdTdT; R: UAUUUGUGCUCUAUUAGCCdTdT |
| hs-CUL5-si-2 | F: CCAGCUGAUUCAGUUAAUAdTdT; R: UAUUAACUGAAUCAGCUGGdTdT |
| hs-DDB2-si-1 | F: CCCAAGAAACGCCCAGAAAdTdT; R: UUUCUGGGCGUUUCUUGGGdTdT |
| hs-DDB2-si-2 | F; GGACCCUCCACCAGCAUAAdTdT; R: UUAUGCUGGUGGAGGGUCCdTdT |
| hs-DCAF7-si-1 | F; ACAAAGAGGUCUAUGAUAUdTdT; R: AUAUCAUAGACCUCUUUGUdTdT |
| hs-DCAF7-si-2 | F; GGAAGGAGAUCUACAAGUAdTdT; R: UACUUGUAGAUCUCCUUCCdTdT |
| hs-DCAF13-si-1 | F; GUCUGCUAGUUUCGAUAAAdTdT; R: UUUAUCGAAACUAGCAGACdTdT |
| hs-DCAF13-si-2 | F: GUGGAAAGCUAAUGCUUCUdTdT; R: AGAAGCAUUAGCUUUCCACdTdT |
