# Supplementary figures and images for: CUL4B-DDB1-COP1-mediated UTX downregulation promotes colorectal cancer progression
Source: Exp Hematol Oncol. 2023 Sep 7;12:77. doi: 10.1186/s40164-023-00440-z (PMC10483726; doi:10.1186/s40164-023-00440-z)

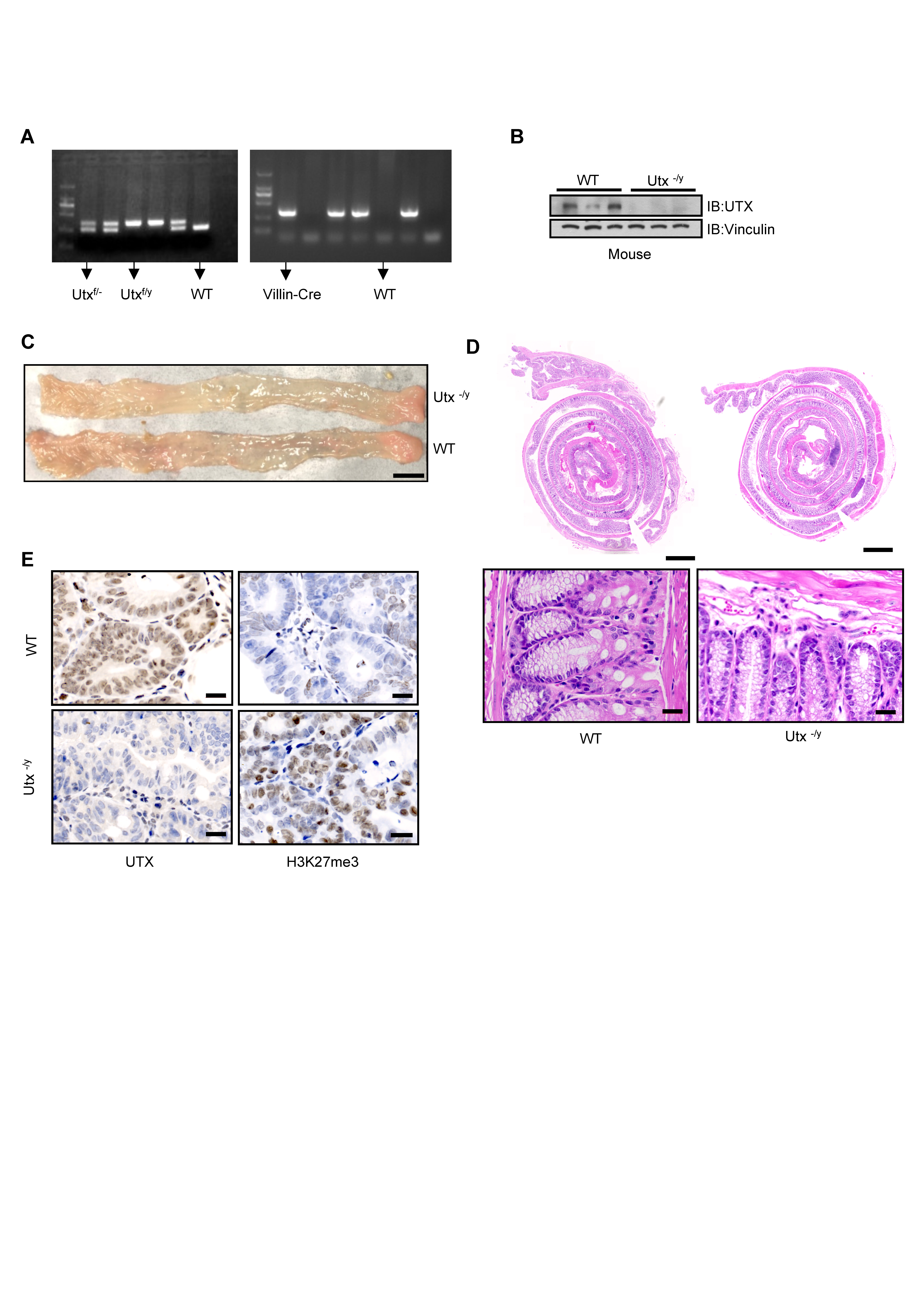

Supplement: Supplementary file 2 — Additional file 2: Figure S1. Loss of UTX contributes to CRC. A Genotyping of Utx and Villin-CRE mice by PCR. B IB analysis of UTX expression in large intestines of WT and Utx-/y mice. C and D Macroscopic image (C) and H&E staining (D) of large intestines derived from 16-month-old WT and Utx-/y mice. Scale bar in (C), 5000 μm. Scale bar in (D), 1000 μm (upper) and 20 μm (bottom). E Representative IHC images of UTX and H3K27me3 staining in large intestines of WT and Utx-/y mice post AOM/DSS-induced CRC tumorigenesis. Scale bar, 20 μm. [file 40164_2023_440_MOESM2_ESM.tif]

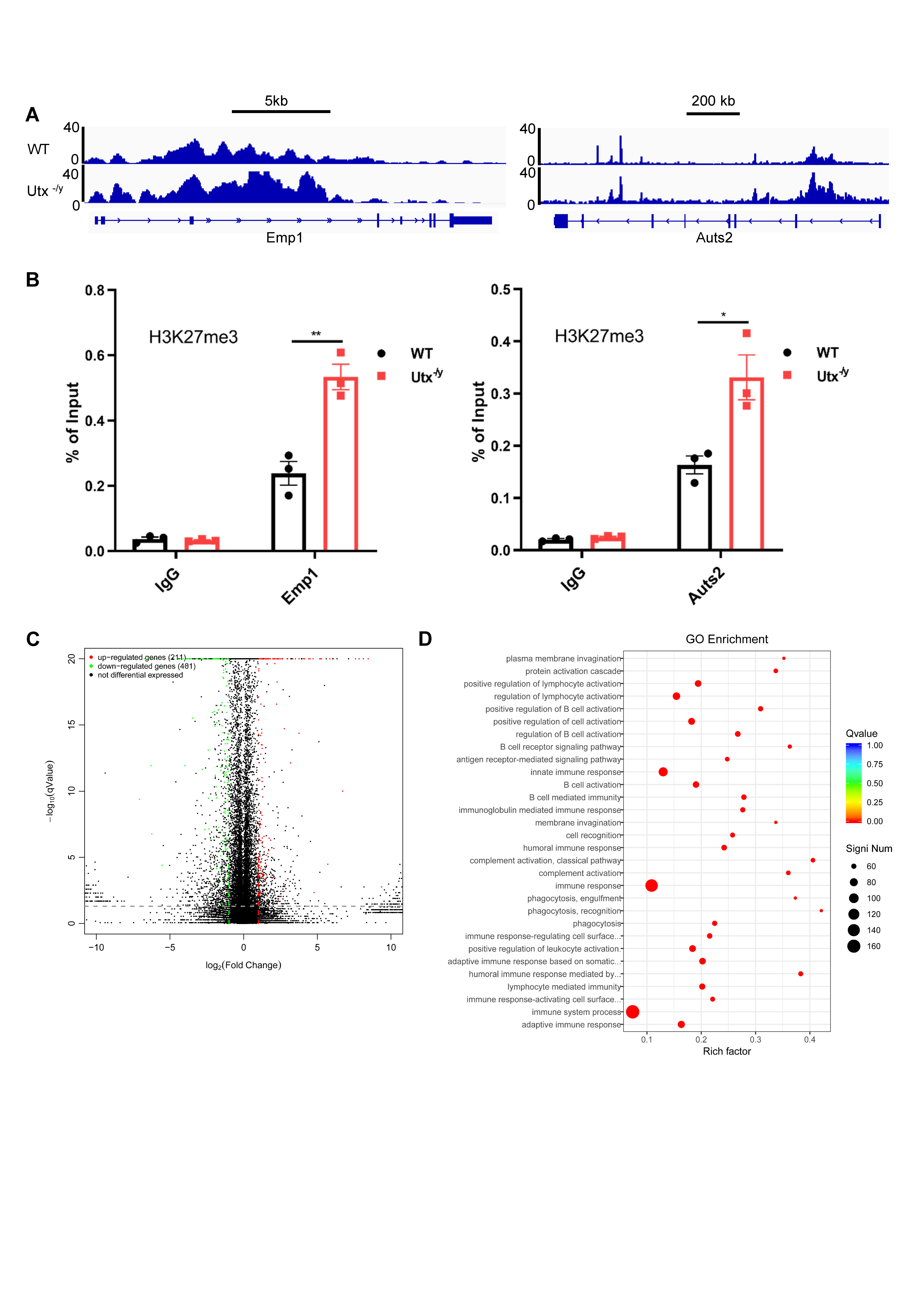

Supplement: Supplementary file 3 — Additional file 3: Figure S2. EMP1 and AUTS2 are putative UTX target genes. A ChIP-seq tracks for H3K27me3 at Emp1 and Auts2 gene locus. B ChIP-qPCR of H3K27me3 modifications on the promoter regions of the indicated genes. C Volcano plots showing the differentially expressed genes in large intestines between WT and Utx-/y mice. D GO enrichment analysis showed biological processes regulated by Utx. Data information: In (B), data are presented as mean±SEM (two-tailed Student’s t-test). *P<0.05, **P<0.01 [file 40164_2023_440_MOESM3_ESM.tif]

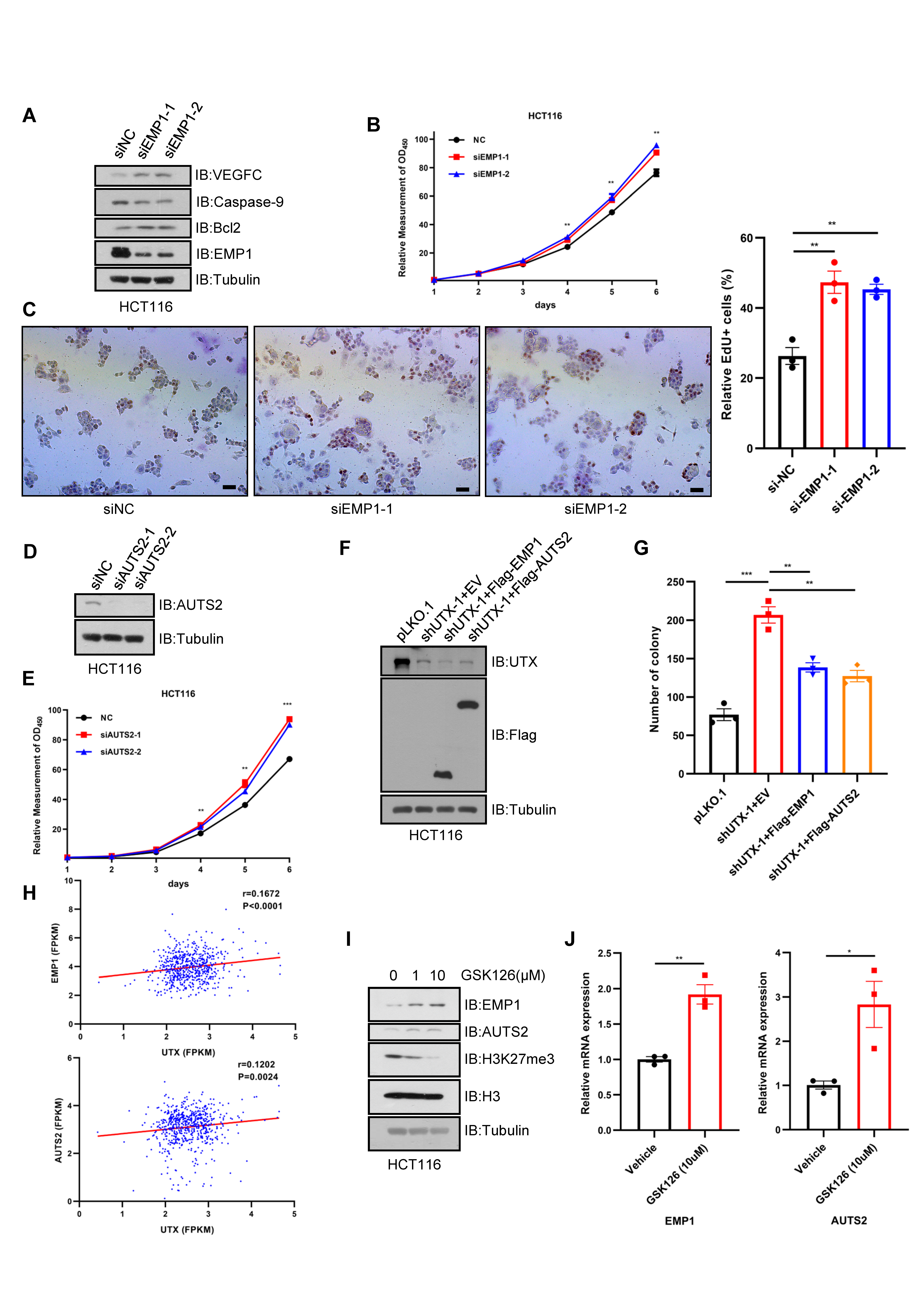

Supplement: Supplementary file 4 — Additional file 4: Figure S3 Functional correlation between UTX and EMP1 or AUTS2. A IB analysis of WCL from HCT116 cells transfected with siEMP1 or NC. B Cell growth curve analysis of HCT116 cells transfected with siEMP1 or NC. C Proliferation of HCT116 cells transfected with indicated siRNA was detected by EdU assay and observed under light microscopy (left). Scale bar, 50 μm. Statistical analysis of EdU-positive cells in different groups (right). D IB analysis of WCL from HCT116 cells transfected with siAUTS2 or NC. E Cell growth curve analysis of HCT116 cells transfected with siAUTS2 or NC. F IB analysis of WCL from HCT116 cells infected with indicated lentiviruses. G Growth of HCT116 cells infected with indicated lentiviruses was detected by the anchorage-independent soft agar assay. Statistical analysis of soft agar assays in different groups. H UTX expression was positively correlated with EMP1 and AUTS2 in the TCGA dataset. I IB analysis of HCT116 cells treated with different concentrations of GSK126 (0 μM, 1 μM, 10 μM). J qPCR analysis of EMP1 and AUTS2 expression in HCT116 cells treated with vehicle or GSK126 (10 μM). Data information: In (B, C, E and J), data are presented as mean±SEM (two-tailed Student’s t-test). In (G), data are presented as mean±SEM (one-way ANOVA with Dunnett’s multiple comparisons test). *P<0.05, **P<0.01, ***P<0.001. [file 40164_2023_440_MOESM4_ESM.tif]

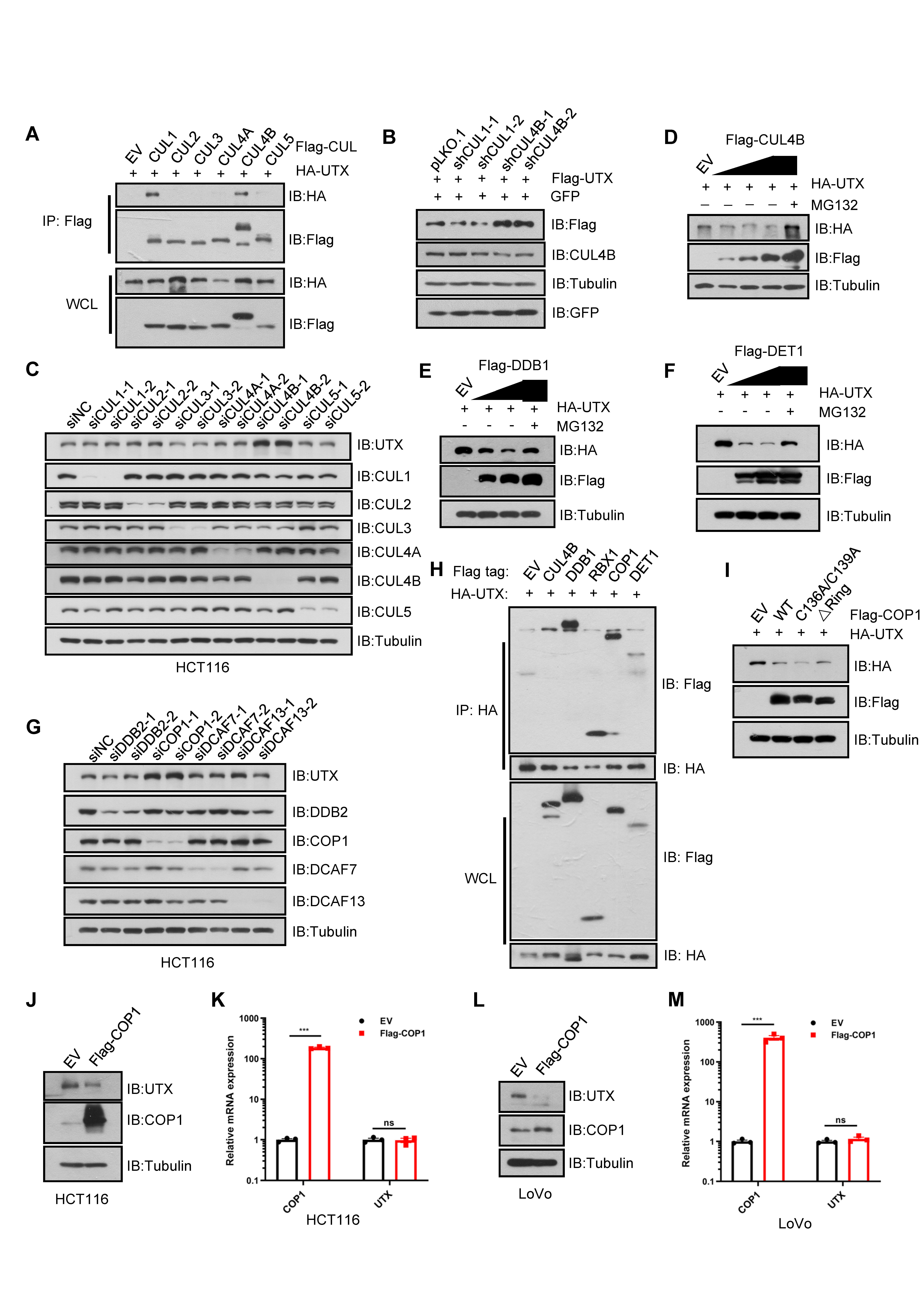

Supplement: Supplementary file 5 — Additional file 5: Figure S4. CRL4-COP1 E3 complex modulates UTX stability. A IB analysis of WCL and IP from HEK293T cells co-transfected with HA-UTX and different Cullin family constructs. Cells were treated with MG132 (20 µM) for 12 h before they were harvested. B IB analysis of WCL from HEK293T cells co-transfected with Flag-UTX and shCUL1 or shCUL4B. C IB analysis of WCL from HCT116 cells transfected with indicated siRNA targeting different Cullins. D IB analysis of WCL derived from HEK293T cells co-transfected with various doses of Flag-CUL4B together with HA-UTX constructs. Cells were treated with or without MG132, as indicated before they were harvested. E IB analysis of WCL derived from HEK293T cells co-transfected with various doses of Flag-DDB1 and HA-UTX constructs. Cells were treated with or without MG132, as indicated before they were harvested. F IB analysis of WCL derived from HEK293T cells co-transfected with various doses of Flag-DET1 and HA-UTX constructs. Cells were treated with or without MG132, as indicated before they were harvested. G IB analysis of WCL from HCT116 cells transfected with indicated siRNA targeting different CUL4B adaptor E3 ligases. H IB analysis of WCL and IP from HEK293T cells co-transfected with HA-UTX and CUL4B complex. Cells were treated with MG132 (20 µM) for 12 h before they were harvested. I IB analysis of WCL derived from HEK293T cells co-transfected with HA-UTX and Flag-COP1 (WT or C136A/C139A or △Ring). J and K IB (H) and qPCR analysis (I) of UTX expression in HCT116 cells infected with lentiviruses expressing EV or Flag-COP1. L and M IB (J) and qPCR analysis (K) of UTX expression in LoVo cells infected with lentiviruses expressing EV or Flag-COP1. Data information: In (K and M), data are presented as mean±SEM (two-tailed Student’s t-test). ns, non-significance, ***P<0.001 [file 40164_2023_440_MOESM5_ESM.tif]

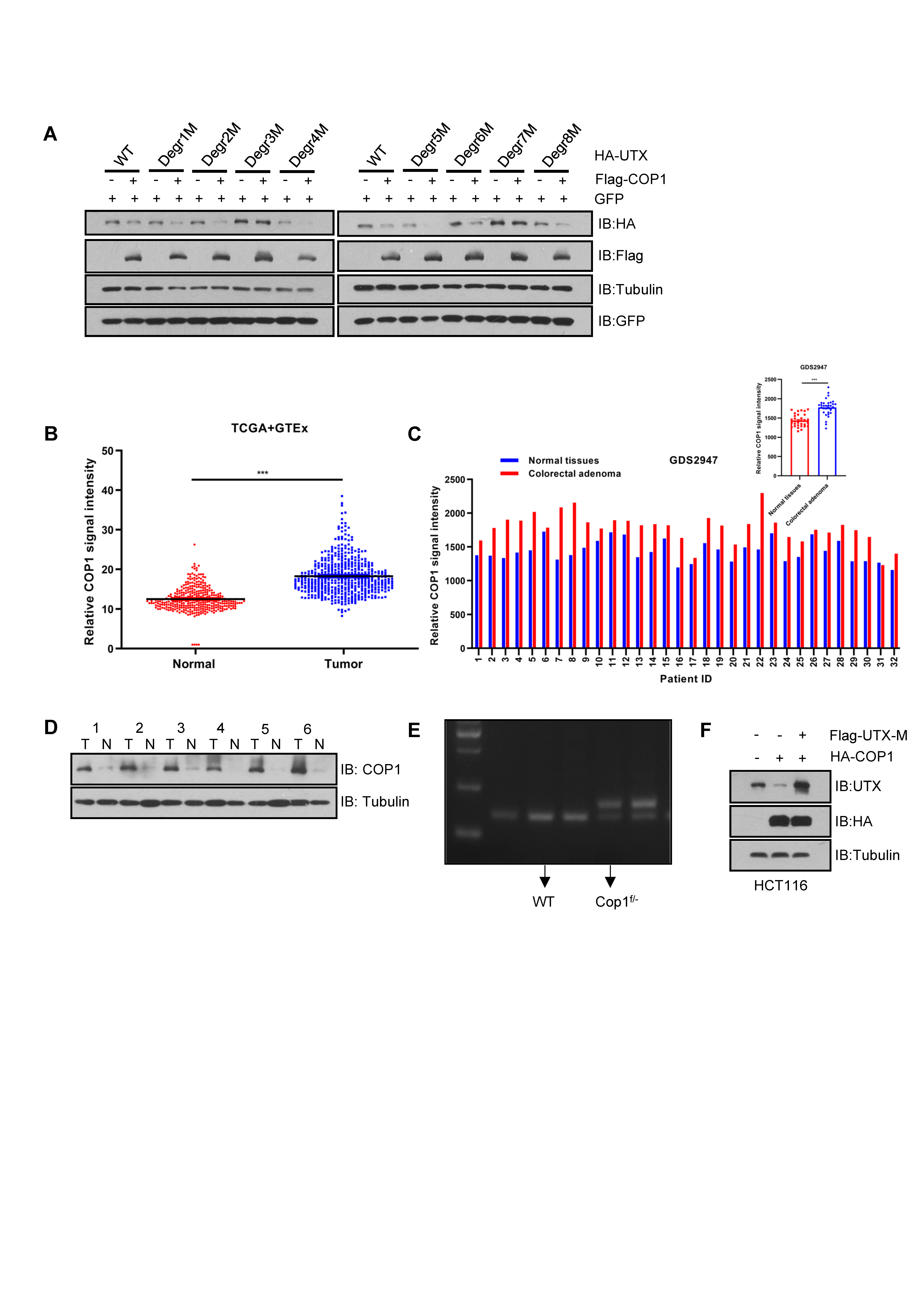

Supplement: Supplementary file 6 — Additional file 6: Figure S5. COP1 is an oncogenic protein in CRC. A IB analysis of WCL from HEK293T cells co-transfected with Flag-COP1 and WT or mutant HA-UTX. B COP1 mRNA levels in colon cancer tissues (n=471) and normal tissues (n=349) were determined from TCGA and GETx databases. C COP1 mRNA levels in colorectal adenoma (n=32) and normal tissues (n=32) were determined from GDS2947. D IB analyses of COP1 expression in six pairs of random CRC samples. T, matched tumor tissues; N, adjacent normal specimens. E Genotyping of Cop1f/- mice by PCR. F IB analysis of WCL from HCT116 cells with ectopic HA-COP1 or Flag-UTX-mutant (V607A/P608A/V1205A/P1206A) expression. Data information: In (B and C), data are presented as mean±SEM (two-tailed Student’s t-test), ***P<0.001 [file 40164_2023_440_MOESM6_ESM.tif]
